# Draft genome of the Antarctic dragonfish, *Parachaenichthys charcoti*

**DOI:** 10.1093/gigascience/gix060

**Published:** 2017-07-24

**Authors:** Do-Hwan Ahn, Seung Chul Shin, Bo-Mi Kim, Seunghyun Kang, Jin-Hyoung Kim, Inhye Ahn, Joonho Park, Hyun Park

**Affiliations:** 1 Unit of Polar Genomics, Korea Polar Research Institute, Incheon 21990, South Korea; 2 Polar Sciences, University of Science and Technology, Yuseong-gu, Daejeon 34113, South Korea; 3 Department of Fine Chemistry, Seoul National University of Science and Technology, Seoul 01811, South Korea

**Keywords:** *Parachaenichthys charcoti*, antarctic dragonfish, notothenioid, *de novo* genome assembly; genome annotation

## Abstract

The Antarctic bathydraconid dragonfish, *Parachaenichthys charcoti*, is an Antarctic notothenioid teleost endemic to the Southern Ocean. The Southern Ocean has cooled to −1.8ºC over the past 30 million years, and the seawater had retained this cold temperature and isolated oceanic environment because of the Antarctic Circumpolar Current. Notothenioids dominate Antarctic fish, making up 90% of the biomass, and all notothenioids have undergone molecular and ecological diversification to survive in this cold environment. Therefore, they are considered an attractive Antarctic fish model for evolutionary and ancestral genomic studies. Bathydraconidae is a speciose family of the Notothenioidei, the dominant taxonomic component of Antarctic teleosts. To understand the process of evolution of Antarctic fish, we select a typical Antarctic bathydraconid dragonfish, *P. charcoti*. Here, we have sequenced, *de novo* assembled, and annotated a comprehensive genome from *P. charcoti*. The draft genome of *P. charcoti* is 709 Mb in size. The N50 contig length is 6145 bp, and its N50 scaffold length 178 362 kb. The genome of *P. charcoti* is predicted to contain 32 712 genes, 18 455 of which have been assigned preliminary functions. A total of 8951 orthologous groups common to 7 species of fish were identified, while 333 genes were identified in *P. charcoti* only; 2519 orthologous groups were also identified in both *P. charcoti* and *N. coriiceps*, another Antarctic fish. Four gene ontology terms were statistically overrepresented among the 333 genes unique to *P. charcoti*, according to gene ontology enrichment analysis. The draft *P. charcoti* genome will broaden our understanding of the evolution of Antarctic fish in their extreme environment. It will provide a basis for further investigating the unusual characteristics of Antarctic fishes.

## Data description

### Introduction

The fish fauna of the Southern Ocean is dominated by a single lineage belonging to the perciform suborder Notothenioidei, consisting of 132 species and 8 families. All Antarctic notothenioids have evolved to adapt to the extreme Antarctic marine environment, which includes large seasonal changes in food availability and stably cold water temperature. Notothenioids dominate Antarctic fish, making up 90% of the biomass, and all notothenioids have undergone molecular and ecological diversification to survive in this cold environment. Therefore, they are considered an attractive Antarctic fish model for evolutionary and ancestral genomic studies. Bathydraconidae is a speciose family of the Notothenioidei, the dominant taxonomic component of Antarctic teleosts [1–4]. *Parachaenichthys charcoti*, the Antarctic bathydraconid dragonfish, was first described by Vaillant in 1906 (Notothenioidei: Bathydraconidae; AphiaID: 234687; Fishbase ID: 7102). They are found in localities around Potter Cove, South Shetland Islands. *P. charcoti* remain almost exclusively on the inner shelves throughout their ontogeny [5]. Several studies have investigated their ecology and ethology, but there has been no genomic study [5–8]. A comprehensive genetic study is needed to identify the distinguishing characteristics of this Antarctic fish and to provide useful data for understanding Antarctic teleost divergence and evolution.

### Library construction and sequencing

**Figure 1:**
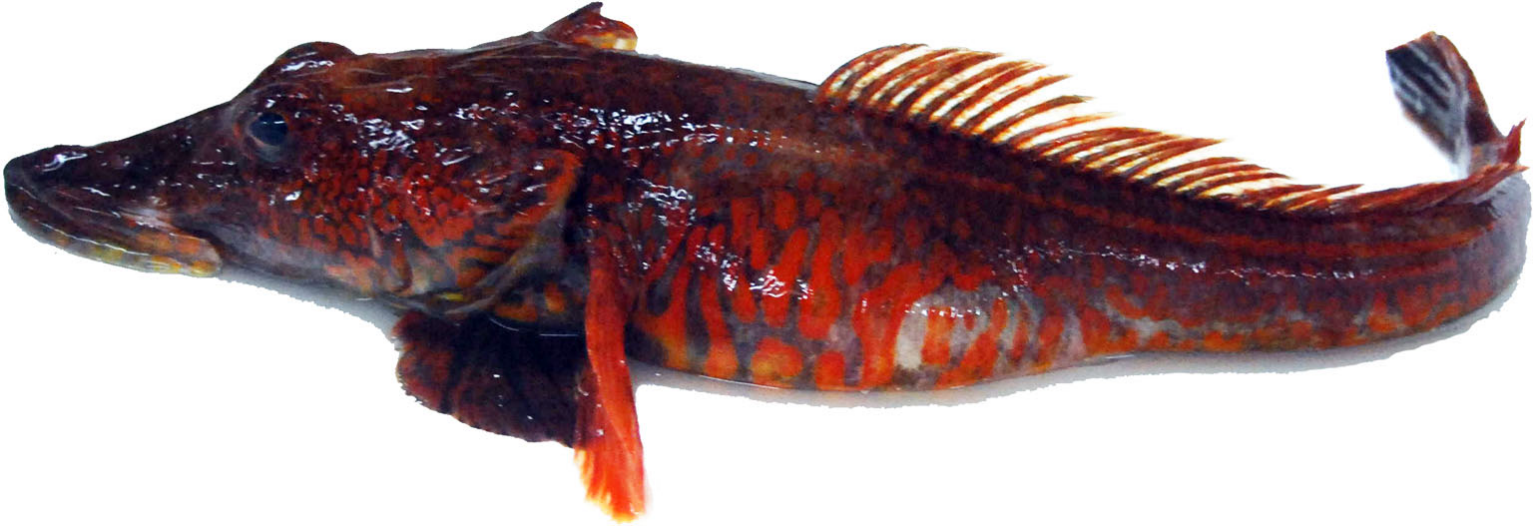
Photograph of Antarctic dragonfish, *P. charcoti.*

*P. charcoti* (length: ∼45 cm) were collected in nets at depths of 20–30 m in Marian Cove, near King Sejong Station, on the Northern Antarctic Peninsula (62°14'S, 58°47'W) in January 2012 using the hook-and-line method (Fig. 1). High–molecular weight genomic DNA was extracted from *P. charcoti* using the Gentra Puregene Blood Kit (Qiagen, Valencia, CA, USA). For genomic DNA sequencing, 3 paired-end libraries (PE300, PE400, and PE450) were constructed from sheared genomic DNA (consisting of 300-, 400-, and 450-bp fragments) and subsequently prepared using standard Illumina sample preparation methods. Mate-pair libraries (MP3K, MP5K, MP8K, and MP20K) were prepared for scaffolding, and sequencing was performed according to the manufacturer's instructions (consisting of 3-kb, 5-kb, 8-kb, and 20-kb fragments; Illumina, San Diego, CA, USA).

Because expressed sequence tags are essential for gene annotation in draft genomes, a transcriptome library was conducted using TruSeq® Sample Preparation v. 2 (Illumina) with total RNA. Total RNA was extracted from liver tissue and purified using the RNeasy Mini Kit (Qiagen) with the RNase-Free DNaseI Kit (Qiagen). Extracted sample quality and concentration were determined with 2100 Bioanalyzer (Agilent Technologies, Santa Clara, CA, USA). mRNA was isolated from 2 μg of the total RNA for double-stranded cDNA library construction with poly-A selection. For transcriptome sequencing, paired-end libraries (PE500) were constructed from sheared cDNA consisting of 500-bp fragments and subsequently prepared using standard Illumina sample preparation methods. Final transcriptome libraries’ length and concentration were determined with 2100 Bioanalyzer. Transcriptome libraries were sequenced using runs of 300 × 2 paired-end reads (Table 1).

**Table 1:** *P. charcoti* sequencing statistics

Library	Mode	Insert size (bp)	Library type	Trimmed reads	Trimmed sequence (bp)	Source
PE300	2 × 300	300	Paired-end	28 776 064	4 964 428 226	Genomic DNA
PE400	2 × 300	400	Paired-end	139 126 700	29 538 419 473	Genomic DNA
PE450	2 × 300	450	Paired-end	85 834 292	16 644 575 781	Genomic DNA
MP3K	2 × 300	3000	Mate-pair	70 517 546	4 925 657 177	Genomic DNA
MP5K	2 × 300	5000	Mate-pair	66 623 428	4 626 486 038	Genomic DNA
MP8K	2 × 300	8000	Mate-pair	61 240 982	4 212 744 363	Genomic DNA
MP20K	2 × 300	20 000	Mate-pair	86 575 644	5 387 730 972	Genomic DNA
PE500	2 × 300	500	Paired-end	25 940 404	5 571 197 784	Liver RNA

All resulting Illumina reads were trimmed using the FASTX-Toolkit (v. 0.0.11) [9] with the parameters -t 20, -l 70, and -Q 33, after which a paired sequence from the trimmed Illumina reads was selected. All sequencing processes for 3 paired-end libraries (genomic DNA), 4 mate-pair libraries (genomic DNA), and 1 paired-end library (transcriptome) were performed by Korea Polar Research Institutes (data statistics provided in Table 1).

### Genome assembly

**Figure 2:**
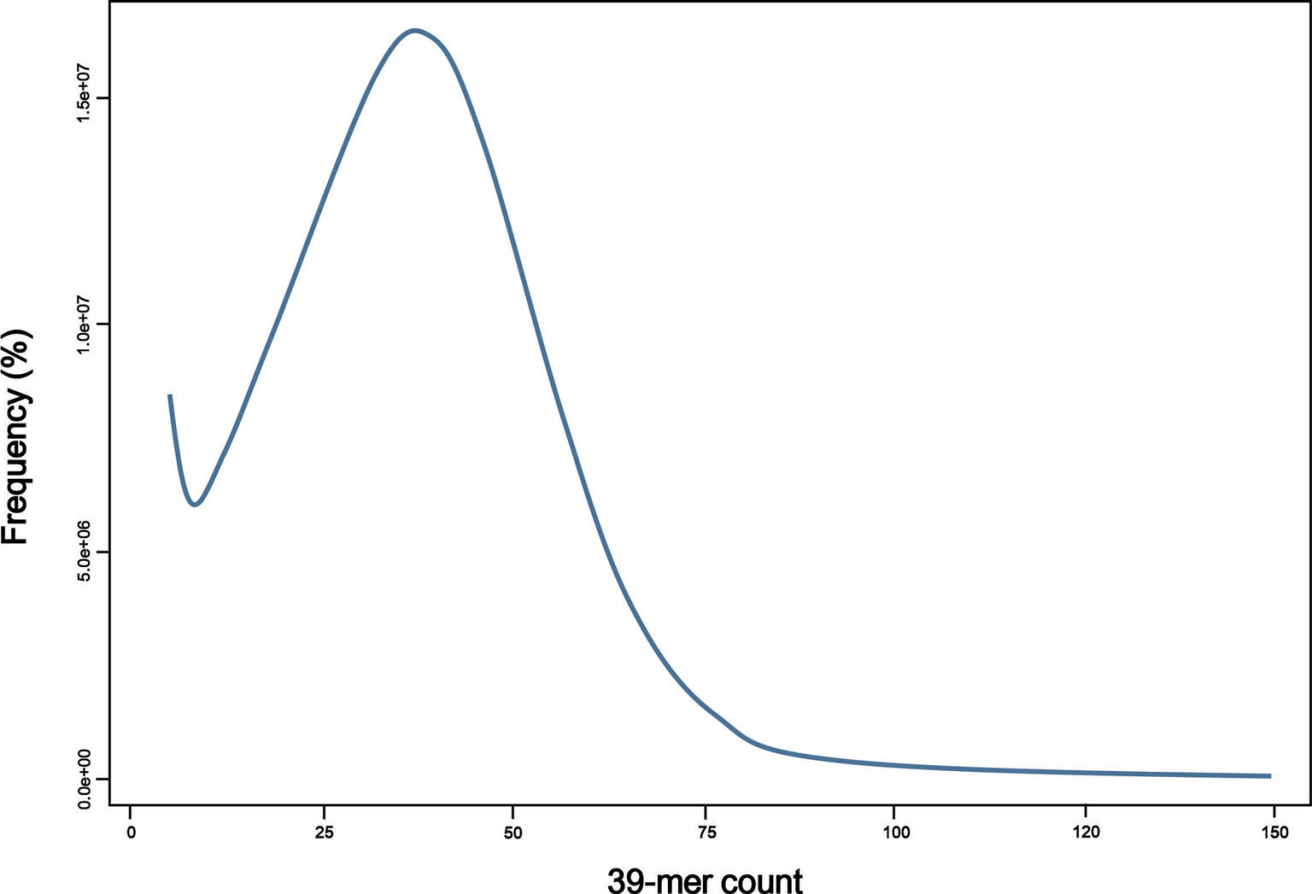
Estimation of the *P. charcoti* genome size based on 39-mer analysis. X-axis represents the depth (peak at ×39), and the y-axis represents the proportion. Genome size was estimated to be 805 Mb (total k-mer number/volume peak).

K-mer analysis was conducted using Jellyfish 2.2.5 (Jellyfish, RRID:SCR_005491) [10] to estimate the genome size from DNA paired-end libraries. The estimated genome size is 805 Mb, with the main peak observed at a coverage depth of ∼×39 (Fig. 2). Initial assemblies were performed using the Celera Assembler v. 8.3 (Celera Assembler, RRID:SCR_010750) with trimmed paired-end reads [11]. For the Celera Assembler, paired-end read data were converted into FRG file format using FastqToCA, which is a utility included in the Celera Assembler. Assembly was performed on an 80-processor workstation using Intel Xeon X7460 2.66 GHz processors and 1 Tb of RAM with the following parameters: overlapper = ovl, unitigger = bogart, utgErrorRate = 0.03, utgErrorLimit = 2.5, utgGraphErrorRate = 0.030, utgGraphErrorLimit = 3.25, ovlErrorRate = 0.06, cnsErrorRate = 0.06, cgwErrorRate = 0.1, merSize = 28, doOverlapBasedTrimming = 1, merylMemory = 500 000, merylThreads = 40, ovlMemory = 8 Gb, ovlThreads = 2, ovlConcurrency = 40, ovlHashBlockLength = 300 000 000, ovlRefBlockSize = 7 630 000, and ovlHashBits = 24. The initial assembly had a total size of 709 Mb, N50 contig length of 5039 bp, and N50 scaffold length of 6135 kb, with a GC content of 40.66%. The assembled contig revealed a contig coverage of approximately ×36.57 from the Celera Assembler. Contigs from the initial assembly were used for scaffolding using the stand-alone scaffolding tool SSPACE v. 2.0 (SSPACE, RRID:SCR_005056) with the following parameters: -x 0, -k 3, -a 0.8, and -T 60 [12].

Trimmed mate-pair reads created using the FASTX-Toolkit were used in the scaffolding process. After scaffolding, the number of scaffolds decreased from 153 398 to 12 381, and the N50 scaffold length increased from 6135 to 166 726 bp (Table 2). The total size of the final scaffolds (∼795 Mb) was consistent with the estimated genome size (805 Mb).

**Table 2:** Global statistics of the *P. charcoti* genome assembly

		*P. charcoti*
Scaffold	Total scaffold length (bases)	794 596 176
	Gap size (bases)	86 840 902
	Scaffolds (*n*)	12 602
	N50 scaffold length (bases)	178 362
	Max scaffold length (bases)	1 318 127
Contig	Total contig length (bases)	709 540 340
	Contigs (*n*)	153 398
	N50 contig length (bases)	6145
	Max contig length (bases)	65 864
Annotation	Gene number (*n*)	32 712
	An average mRNA length (bases)	1412
	An average CDS length (bases)	1291
	An average of exons (*n*)	8
Repeat content (% of genome)		19.4

### Gene annotation

MAKER2 annotation pipeline (MAKER, RRID:SCR_005309) was used for genome annotation with default parameters [13]. It first identified repetitive elements using RepeatMasker v. 3.3.0 (Repeat Masker, RRID:SCR_012954) with a *de novo* repeat library [14], which was constructed using RepeatModeler v. 1.0.3 (RepeatModeler, RRID:SCR_015027) [15] with the Repbase library (Ver. 20 140 131). The SNAP gene finder [16] was selected to perform *ab initio* gene prediction from this masked genome sequence. Alignment of transcriptome assembly results using BLASTn and homologous protein information from tBLASTx were considered for gene annotation as RNA and protein evidence, respectively. Transcriptome assembly was performed by using the program CLC Genomics Workbench 8.0 with default parameters, and sequencing reads from PE500 (Table 1) were used. Proteins from 6 species were used in the analysis: *Notothenia coriiceps* (NCBI reference sequence NC_015653.1) and *Danio rerio, Gasterosteus aculeatus, Takifugu rubripes, Tetraodon nigroviridis*, and *Gadus morhua* (all from Ensembl release 69). MAKER2 includes integration of the annotation edit distance (AED) metric for controlling the quality of annotation [17]. AED values are bounded between 0 and 1; an AED value of 0 indicated that its aligned evidence and annotated gene showed an exact match. Conversely, a value of 1 indicated no evidence support. But the AED cut-off was not applied for these gene predictions. Instead, AED values were denoted in gene annotation and were considered for orthologous gene analysis and gene gain and loss.

MAKER2 was used to select and revise the final gene model based on all inputs. A total of 32 712 genes were predicted in *P. charcoti* using MAKER2 (Table 2). The annotated genes contained an average of 8 exons, with an average mRNA length of 1412 bp and coding DNA sequences (CDS) length of 1291 bp. The repeat prediction from MAKER2 showed that repeat sequences accounted for 19.41% of the assembled *P. charcoti* genome.

To estimate genome assembly and annotation completeness, we performed Benchmarking Universal Single-Copy Orthologs (BUSCO) analysis (BUSCO, RRID:SCR_015008) [18], an approach used for lineage-specific profile libraries, such as those of actinopterygii, and identified 88.6% complete and 5.7% partial eukaryote orthologous gene sets in our assembly (Table 3).

**Table 3:** Summarized benchmarks of the BUSCO assessment

	Actinopterygii (%)
Total BUSCO groups searched	4062^a^
Complete BUSCOs	88.6
Complete and single-copy	86.3
Complete and duplicated	2.3
Partial	5.7
Missing	5.7

^*a*^Number of total BUSCO groups searched.

To assign preliminary functions for 32 712 genes, we used Blast2GO v. 2.6.0 (Blast2GO, RRID:SCR_005828) [19]. We classified functions for 18 455 (56.42%) predicted genes, which were annotated using BLASTp results and InterproScan (RRID:SCR_005829). Gene ontology annotation terms included “biological process” (20 126, 61.52%), “molecular function” (20 514, 62.71%), and “cellular component” (15 452, 47.23%). Enzyme commission numbers were obtained for 3846 proteins.

### Ortholog analysis

**Figure 3:**
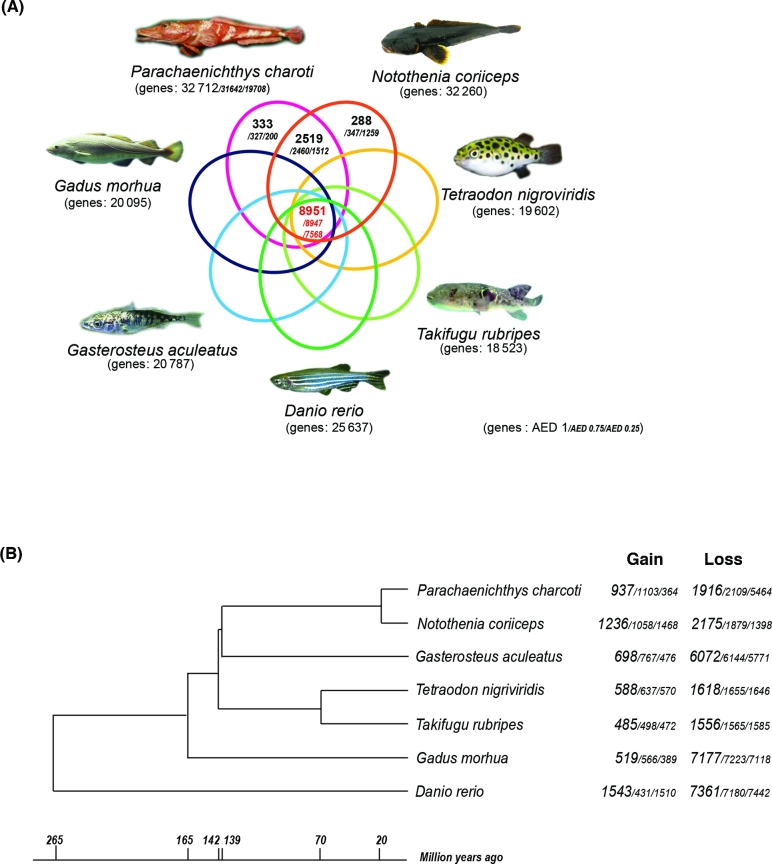
Comparative genome analyses of the *P. charcoti* genome. (**A**) Venn diagram of orthologous gene clusters between 4 arthropod lineages. (**B**) Gene family gain-and-loss analysis. The number of gained gene families and lost gene families are indicated for each species. Time lines specify divergence times between the lineages.

We identified orthologous groups using OrthoMCL (v. 2.0.5) [20], which generated a graphical representation of the sequence relationships, which were then presented in subgraphs using the Markov Clustering Algorithm based on multiple eukaryotic genomes. We used the standard parameters (percentMatchCutoff = 50 and evalueExponentCutoff = –5) and options within OrthoMCL for all steps. We used 7 fish genomes for this analysis (*D. rerio, G. aculeatus, T. rubripes, T. nigroviridis, G. morhua, N. coriiceps*, and *P. charcoti*). The coding sequences of 5 genomes were collected from Ensembl release 69, and 1 coding sequence was selected among multiple proteins corresponding to 1 gene. We used the coding sequence from the NCBI reference sequence (NC_015653.1) of *N. coriiceps* and 3 groups of the coding sequence of *P. charcoti* from MAKER annotation with different AED thresholds (1, 0.75, and 0.25). In the case of a AED cut-off value of 1, we identified 8951 orthologous groups common to all 7 fish; 288 of 32 636 *N. coriiceps* genes and 333 of 32 712 *P. charcoti* genes were not identified in any other species, and 2519 groups were identified only in the 2 Antarctic fish (Fig. 3A). When we applied an AED threshold of 0.25 against gene prediction of *P. charcoti*, 7568 orthologous groups were identified.

### Likelihood analysis of gene gain and loss

We estimated differences in the size of orthologs to identify gene families that have undergone significant size changes through evolution [21, 22]. We used the program CAFE3.0 [23] and performed analyses against 3 groups including the coding sequence of *P. charcoti* with different AED thresholds separately. We performed phylogenetic analyses among 7 representative fishes with the protein-coding gene in the orthologous groups to obtain the Newick description of a rooted and bifurcating phylogenetic tree. A total of 8951 orthologous gene sets were selected using the criterion of reciprocal best BLASTP hit and were aligned using PRANK (v. 130820) under a codon model with the “-dna -codon” option [24]; poor alignment sites were eliminated using Gblock (v. 0.91) under a codon model with the “-t = c” option [25]. The remaining alignment regions were concatenated and used in the construction of the phylogenetic tree by using the neighbor-joining method in the MEGA (v. 6) program (MEGA, RRID:SCR_000667) [26]. The ultrametric tree of the species with branch lengths in units of time was prepared by referring TimeTree [27] for CAFE3.0 (Fig. 3B). The program was performed using *P* < 0.05, and estimated rates of birth (λ) and death (μ) were calculated using the program LambdaMu with the “-s” option. The numbers of gene gains and losses were calculated on each branch of the tree with the “-t” option. *P. charcoti* gained 937 and lost 1916 gene families (Fig. 3B).

The Antarctic dragonfish *P. charcoti* is a species in the sister lineage of icefishes [28–30]; it is the only hemoglobinless vertebrate. The dragonfish (Bathydraconidae) and the icefish (Channichthyidae) were generally considered to have evolved from a common notothenioid ancestor, which was characterized by decreased hematocrit and blood hemoglobin concentrations [31–35]. The dragonfish showed the most similar patterns in these trends among red-blooded notothenioid taxa [35]. The globin complex of the dragonfish *P. charcoti* was hypothesized to be similar in length and organization to that of ancestral icefish prior to loss of functionality [36]. Along with the recently published *N. coriiceps* genome [37], the genome of *P. charcoti* will broaden our understanding of how Antarctic fish have evolved to survive in sub-zero temperatures and might provide an important clue to understand the process of evolution to the hemoglobinless Antarctic fish and their distinct phenotypes (an increase of blood volume, low blood viscosity, large bore capillaries, increased vascularity with great capacitance, cardiomegaly, and high blood flow).

## Availability of supporting data

The data for the *P. charcoti* genome and transcriptome have been deposited in the Sequence Read Archive as BioProject PRJNA330735. Other supporting data, including annotations, alignments, and BUSCO results, are available in the *GigaScience* repository, *Giga*DB [38].

## Abbreviations

AED: annotation edit distance; BUSCO: Benchmarking Universal Single-Copy Orthologs; CDS: coding DNA sequences.

## Competing interests

The authors declare no competing interests.

## Funding

This work was supported by a grant entitled “Polar Genomics 101 Project (PE17080),” funded by the Korea Polar Research Institute.

## Author contributions

H.P. and J.P. conceived and designed experiments and analyses. D.H.A., S.C.S., B.K., S.K., J.K., I.A., and J.P. performed experiments and conducted bioinformatics. D.H.A., S.C.S., B.K., and H.P. wrote the paper.

## Supplementary Material

GIGA-D-17-00041_Original-Submission.pdf

GIGA-D-17-00041_Revision-1.pdf

Response-to-Reviewer-Comments_Original-Submission.pdf

Reviewer-1-Report-(Original-Submission).pdf

Reviewer-1-Report-(Revision-1).pdf

Reviewer-2-Report-(Original-Submission).pdf
