# Supplementary material for: Draft genome of the Antarctic dragonfish, Parachaenichthys charcoti
Source: Gigascience. 2017 Jul 24;6(8):1–6. doi: 10.1093/gigascience/gix060 (PMC5597851; doi:10.1093/gigascience/gix060)
Supplement: GIGA-D-17-00041_Original-Submission.pdf [file gix060_giga-d-17-00041_original-submission.pdf]

Manuscript

Click here to download Manuscript Antarctic dragonfish-V6-3.docx

Draft genome of the Antarctic dragonfish, *Parachaenichthys charcoti*

Do-Hwan Ahn [1†], Seung Chul Shin[1†], Bo-Mi Kim [1†], Seunghyun Kang [1], Jin-Hyoung Kim [1], Inhye Ahn [1,2], Joonho Park[3], Hyun Park [1,2,*]

[1] *Unit of Polar Genomics, Korea Polar Research Institute, Incheon 21990, South Korea*

[2] *Polar Sciences, University of Science & Technology, Yuseong-gu, Daejeon 34113, South Korea*

[3] *Department of Fine Chemistry, Seoul National University of Science and Technology, Seoul 01811 South Korea*

[*]Corresponding authors:

Unit of Polar Genomics, Korea Polar Research Institute, Incheon 21990, South Korea; E-mail address: hpark@kopri.re.kr (H. Park)

[†]These authors contributed equally to this work.

Abstract

Background

The Antarctic bathydraconid dragonfish, *Parachaenichthys charcoti*, is an Antarctic notothenioid teleost endemic to the Southern Ocean. The Southern Ocean has cooled to −1.8C over the past 30 million years, and *P. charcoti* has undergone molecular and ecological diversification to survive in this cold environment. However, little is known about the biology of this species, except that globin intergenic regulatory regions play a role in its low levels of alpha-globin expression. To understand the process of evolution of Antarctic fish, we sequenced and annotated the *P. charcoti* genome.

Findings

The draft genome of *P. charcoti* is 709 Mb in size. The N50 contig length is 6,246 bp and its N50 scaffold length 166,726 kb, with a GC content of 40.66%. The genome of *P. charcoti* is predicted to contain 32,712 genes, 18,455 of which have been assigned preliminary functions. A total of 8,951 orthologous groups common to seven species fish were identified, while 333 genes were identified in *P. charcoti* only; 2,519 orthologous group were also identified in both *P. charcoti* and *N. coriiceps*, another Antarctic fish. Four gene ontology (GO) terms were statistically overrepresented among the 333 genes unique to *P. charcoti*, according to GO enrichment analysis.

Conclusions

The draft *P. charcoti* genome will broaden our understanding of the evolution of Antarctic fish in their extreme environment. It will provide a basis for further investigating the unusual characteristics of Antarctic fishes.

Keywords

Parachaenichthys charcoti, Antarctic fish, Southern Ocean.

Data description

Antarctic notothenioid teleosts have evolved to adapt to the extreme Antarctic marine environment. The fish fauna of the Southern Ocean is dominated by a single lineage belonging to the perciform suborder Notothenioidei, consisting of 132 species and 8 families. They survive in the extreme Antarctic marine environment, which includes large seasonal changes in food availability and cold ocean water. Nototheniidae is the most speciose family of the Notothenioidei, the dominant taxonomic component of Antarctic teleosts, making up 90% of the fish biomass of the continental shelf and upper slope [1–4]. *Parachaenichthys charcoti*, the Antarctic bathydraconid dragonfish, was first described by Vaillant in 1906. They are found in localities around Potter Cove, South Shetland Islands. *Parachaenichthys charcoti* remain almost exclusively on the inner shelves throughout their ontogeny [5]. Several studies have investigated their ecology and ethology, but there has been no genomic study [5-8]. A comprehensive genetic study is needed to identify the distinguishing characteristics of this Antarctic fish and to provide useful data for understanding Antarctic teleost divergence and evolution.

Library construction and Sequencing

P. charcoti (length: ~45 cm) were collected in nets at depths of 20–30 m in Marian Cove, near King Sejong Station, on the Northern Antarctic Peninsula (62°14'S, 58°47'W) in January 2012 using the hook-and-line method (Fig. 1). High-molecular-weight genomic DNA was extracted from *P. charcoti* using the Gentra Puregene Blood Kit (Qiagen). For Illumina MiSeq sequencing, three paired-end libraries were constructed from sheared genomic DNA consisting of 300, 400, and 450 bp fragments and subsequently prepared using standard Illumina sample

preparation methods. Mate-pair libraries (3 kb, 5 kb, 8 kb, and 20 kb) were prepared for scaffolding, and sequencing was performed according to the manufacturer's instructions (illumine, Carlsbad, USA). All sequencing processes for both paired-end and mate-pair libraries were performed by Korea Polar Research Institutes (Table 1).

Table 1. *P. charcoti* sequencing statistics.

Library	Mode	Insert size	Library type	Trimmed Reads	Trimmed sequence (bp)	Source
PE300	2 x 300	300	paired-end	28,776,064	4,964,428,226	Genomic DNA
PE400	2 x 300	400	paired-end	139,126,700	29,538,419,473	Genomic DNA
PE450	2 x 300	450	paired-end	85,834,292	16,644,575,781	Genomic DNA
MP3K	2 x 300	3,000	mate-pair	70,517,546	4,925,657,177	Genomic DNA
MP5K	2 x 300	5,000	mate-pair	66,623,428	4,626,486,038	Genomic DNA
MP8K	2 x 300	8,000	mate-pair	61,240,982	4,212,744,363	Genomic DNA
MP20K	2 x 300	20,000	mate-pair	86,575,644	5,387,730,972	Genomic DNA
PE500	2 x 300	500	paired-end	25,940,404	5,571,197,784	Liver RNA

The resulting Illumina reads were trimmed using the FASTX-Toolkit (ver. 0.0.11) (http://hannonlab.cshl.edu/fastx_toolkit) with the parameters -t 20, -l 70, and -Q 33, after which a paired sequence from the trimmed Illumina reads was selected. Finally, paired-end trimmed reads data with 73-fold coverage were obtained (Table 1).

Because expressed sequence tags are essential for gene annotation in draft genomes, Total RNA were extracted from liver tissue and purified using the RNeasy Mini Kit (Qiagen, Valencia, CA) with the RNase-Free DNaseI Kit (Qiagen). Extracted sample quality and concentration were determined with 2100 Bioanalyzer (Agilent Technologies, Santa Clara, CA). Next-generation sequencing (NGS) library was conducted using TruSeq® Sample Preparation v2 (Illumina, San Diego, CA) with total RNA. mRNA was isolated from 2 μg of the total RNA for double-stranded cDNA library construction with poly-A selection. Final transcriptome libraries length

and concentration were determined with 2100 Bioanalyzer. Transcriptome libraries were sequenced by the MiSeq® System (Illumina, San Diego, CA) platform using sequenced runs of 300×2 paired-end reads. Finally, 38,071,794 raw sequencing reads (5,571 Mb) were generated in *P. charcoti* liver transcriptome libraries (Table 1).

Genome assembly

K-mer analysis was conducted using Jellyfish 2.2.5 [9] to estimate the genome size from DNA paired-end libraries. The estimated genome size is 805 Mb, with the main peak observed at a coverage depth of ~39x (Fig. 2). Initial assemblies were performed using the Celera Assembler (ver. 8.3) with trimmed paired-end reads [10]. For the Celera Assembler, paired-end read data were converted into FRG file format using FastqToCA, which is a utility included in the Celera Assembler. Assembly was performed on a 80-processor workstation using Intel Xeon X7460 2.66 GHz processors and 1 Tb RAM with the following parameters: overlapper = ovl, unitigger = bogart, utgErrorRate = 0.03, utgErrorLimit = 2.5, utgGraphErrorRate = 0.030, utgGraphErrorLimit = 3.25, ovlErrorRate = 0.06, cnsErrorRate = 0.06, cgwErrorRate = 0.1, merSize = 28, doOverlapBasedTrimming = 1, merylMemory = 500000, merylThreads = 40, ovlMemory = 8 Gb, ovlThreads = 2, ovlConcurrency = 40, ovlHashBlockLength = 300000000, ovlRefBlockSize = 7630000, and ovlHashBits = 24. The initial assembly had a total size of 709 Mb, N50 contig length of 5,039 bp, and N50 scaffold length of 6,135 kb with a GC content of 40.66%. The assembled contig revealed a contig coverage of approximately 36.57x. Contigs from the initial assembly were used for scaffolding using the stand-alone scaffolding tool SSPACE (ver. 2.0) [11]. Trimmed mate-pair reads created using the FASTX-Toolkit were used in the scaffolding process. After scaffolding, the number of scaffolds decreased from 153,398 to 12,381, and the N50 scaffold length increased from 6,135 to 166,726 bp (Table 2).

Gene annotation

MAKER was used for genome annotation [12]. We first identified repetitive elements using RepeatMasker (ver. 3.3.0) [13], and this masked genome sequence was used for *ab initio* gene prediction using the SNAP software [14]. Alignment of expressed sequence tags using BLASTn and homologous protein information from tBLASTx were considered for gene annotation. Proteins from six species were used in the analysis: *Notothenia coriiceps* (NCBI reference sequence NC_015653.1) and *Danio rerio*, *Gasterosteus aculeatus*, *Takifugu rubripes*, *Tetraodon nigroviridis*, and *Gadus morhua* (all from Ensembl release 69). Transcriptome assembly results, which were generated using CLC Genomics Workbench 8.0, were used for expressed sequence tags.

MAKER was used to select and revise the final gene model based on all inputs. A total of 32,712 genes were predicted in *P. charcoti* using MAKER, and 61,709 *ab initio* predictions with insufficient evidence were generated (Table 2). The annotated genes contained an average of eight exons, with an average mRNA length of 1,412 bp and CDS length of 1,291 bp. The repeat prediction from MAKER showed that repeat sequences accounted for 19.41% of the assembled *P. charcoti* genome.

To estimate genome assembly and annotation completeness, we performed BUSCO (Benchmarking Universal Single-Copy Orthologs) [15] analysis, an approach used for lineage-specific profile libraries, such as those of eukaryotes, metazoans, and vertebrates, and identified 88.4% complete and 6.3% partial eukaryote orthologous gene sets in our assembly (Table 3). In the metazoan gene set, 90.4% complete and 6.5% partial genes were identified, and in the vertebrate gene set, 81.8% complete and 13.5% partial genes were identified.

To assign preliminary functions for 32,712 genes, we used Blast2GO (ver. 2.6.0) [16]. We classified functions for 18,455 (56.42%) predicted genes, which were annotated using BLASTp results and InterproScan. Gene ontology (GO) annotation terms included "biological process"

(20,126, 61.52%), “molecular function” (20,514, 62.71%), and “cellular component” (15,452, 47.23%). Enzyme commission numbers were obtained for 3,846 proteins.

Table 2. Global statistics of the *P. charcoti* genome assembly.

		P. charcoti
Scaffold	Total scaffold length (bases)	794596176
	Gap size (bases)	86840902
	Scaffolds (n)	12602
	N50 scaffold length (bases)	178362
	Max scaffold length (bases)	1318127
Contig	Total contig length (bases)	709540340
	Contigs (n)	153398
	N50 contig length (bases)	6145
	Max contig length (bases)	65864
Gene Number (n)		32712
Repeat content (% of genome)		19.4

Table 3. Summarized benchmarks of the BUSCO (Benchmarking Universal Single-Copy Orthologs) assessment.

	**Eukaryote (%)**	**Metazoa (%)**	**Vertebrate (%)**
Total BUSCO groups searched	303*	978*	2586*
Complete BUSCOs	88.4	90.4	81.8
Complete and single-copy	87.1	87.7	80.6
Complete and duplicated	1.3	2.7	1.2
Fragmented	6.3	6.5	13.5
Missing	5.3	3.1	0.7

* Number of total BUSCO groups searched

Ortholog analysis

We identified orthologous groups using OrthoMCL (ver. 2.0.5) [17], which generated a

graphical representation of the sequence relationships, which were then presented in subgraphs using the Markov Clustering Algorithm based on multiple eukaryotic genomes. We used the standard parameters and options within OrthoMCL for all steps. We used seven fish genomes for this analysis (*D. rerio*, *G. aculeatus*, *T. rubripes*, *T. nigroviridis*, *G. morhua*, *N. coriiceps*, and *P. charcoti*), and the coding sequences of five genomes were collected from Ensembl release 69. We used the coding sequence from the NCBI reference sequence (NC_015653.1) of *N. coriiceps* and the coding sequence of *P. charcoti* from MAKER annotation. We identified 8,951 orthologous groups common to all seven fish; 288 of 32,636 *N. coriiceps* genes and 333 of 32,712 *P. charcoti* genes were not identified in any other species, and 2,519 groups were identified only in the two Antarctic fish (Fig. 3A). Subsequently, gene gain-and loss was analyzed in seven representative fish species, *P. charcoti* gained 937 and lost 1916 gene families (Fig. 3B).

GO enrichment test

We analyzed the 333 genes that were identified only in *P. charcoti* for statistically overrepresented GO terms [18]. We used AgriGO [14], a web-based tool for GO analysis, with a significance threshold of $P = 0.05$. We examined the complete hierarchies of GO terms for each gene. Four GO terms were identified by GO enrichment analysis (Table 4) within the category of “biological process”: “organic substance/metabolic process” (GO:0071704), “peptidyl-tyrosine phosphorylation” (GO:0018108), “peptidyl-tyrosine modification” (GO:0018212), and “cell communication” (GO:0007154). Orthologous groups in the two Antarctic fish were also analyzed, and 16 GO terms in the “biological process” category were statistically overrepresented (Table 5).

Table 4. GO terms statistically overrepresented in *P. charcoti* only.

GO ID	GO tree	GO term	Gene number	P-value	FDR

GO:0071704	P	organic substance metabolic process	13	5.00E-06	0.0069
GO:0018108	P	peptidyl-tyrosine phosphorylation	38	5.50E-05	0.023
GO:0018212	P	peptidyl-tyrosine modification	38	6.60E-05	0.023
GO:0007154	P	cell communication	173	5.20E-05	0.023

Table 5. GO terms statistically overrepresented in the two Antarctic fish only.

GO ID	GO tree	GO term	Gene number	P-value	FDR
GO:0031497	P	chromatin assembly	9	2.80E-06	8.90E-05
GO:0006334	P	nucleosome assembly	9	2.80E-06	8.90E-05
GO:0065004	P	protein-DNA complex assembly	9	2.80E-06	8.90E-05
GO:0034728	P	nucleosome organization	9	2.80E-06	8.90E-05
GO:0006333	P	chromatin assembly or disassembly	9	2.80E-06	8.90E-05
GO:0006323	P	DNA packaging	9	2.80E-06	8.90E-05
GO:0071103	P	DNA conformation change	9	8.20E-05	0.0022
GO:0034622	P	cellular macromolecular complex assembly	14	0.00016	0.0039
GO:0006184	P	GTP catabolic process	11	0.0004	0.0084
GO:0046039	P	GTP metabolic process	11	0.00057	0.011
GO:0034621	P	cellular macromolecular complex subunit organization	14	0.0009	0.015
GO:0051276	P	chromosome organization	12	0.0016	0.025
GO:0006325	P	chromatin organization	10	0.0019	0.028
GO:0000723	P	telomere maintenance	3	0.0024	0.03
GO:0032200	P	telomere organization	3	0.0024	0.03
GO:0051258	P	protein polymerization	5	0.0037	0.043

In this study, we report the annotated draft genome sequence for *P. charcoti*. Along with the recently published *N. coriiceps* genome [19], this new genomic information will broaden our understanding of how Antarctic fish have evolved to survive in sub-zero temperatures.

Availability of supporting data

The data for *P. charcoti* genome and transcriptome has been deposited in the PRJNA330735.

Competing interests

The authors declare no competing interests.

Funding

This work was supported by a grant, entitled ‘Polar Genomics 101 Project (PE17080)’ funded by the Korea Polar Research Institute.

Author contributions

H.P. conceived and designed experiments and analyses. D. H. A., S.C.S., B.K., S.K., J.K., I.A. and J.P. performed experiments and conducted bioinformatics. D. H. A., S.C.S., B.K. and H.P. wrote the paper.

References

1. Eastman JT, Pratt D, Winn W. *Antarctic fish biology: evolution in a unique environment*. Academic Press San Diego; 1993.
2. Eastman JT, Clarke A. A comparison of adaptive radiations of Antarctic fish with those of nonAntarctic fish. *Fishes of Antarctica*. Springer; 1998, p. 3-26.

3. Eastman JT. Antarctic notothenioid fishes as subjects for research in evolutionary biology. *Antarctic Science*. 2000;**12**:276-87.

4. Eakin RR, Eastman JT, Near TJ. A new species and a molecular phylogenetic analysis of the Antarctic fish genus Pogonophryne (Notothenioidei: Artedidraconidae). *Copeia*. 2009;**4**:705-13.

5. Casaux R, Mazzotta A, Barrera-Oro E. Seasonal aspects of the biology and diet of nearshore nototheniid fish at Potter Cove, South Shetland Islands, Antarctica. *Polar Biology*. 1990;**11**:63-72.

6. Barrera-Oro E. The role of fish in the Antarctic marine food web: differences between inshore and offshore waters in the southern Scotia Arc and west Antarctic Peninsula. *Antarctic Science*. 2002;**14**:293-309.

7. Barrera-Oro ER, Lagger C. Egg-guarding behaviour in the Antarctic bathydraconid dragonfish Parachaenichthys charcoti. *Polar biology*. 2010;**33**:1585-7.

8. Eastman JT, Sidell BD. Measurements of buoyancy for some Antarctic notothenioid fishes from the South Shetland Islands. *Polar Biology*. 2002;**25**:753-60.

9. Marçais G, Kingsford C. A fast, lock-free approach for efficient parallel counting of occurrences of k-mers. *Bioinformatics*. 2011;**27**:764-770.

10. Myers EW, Sutton GG, Delcher AL, Dew IM, Fasulo DP, et al. A whole-genome assembly of *Drosophila. Science*. 2000;**287**: 2196-2204.

11. Boetzer M, Henkel CV, Jansen HJ, Butler D, Pirovano W. Scaffolding pre-assembled contigs using SSPACE. *Bioinformatics*. 2011;**27**:578-579.

12. Cantarel BL, Korf I, Robb SM, Parra G, Ross E, et al. MAKER: an easy-to-use annotation pipeline designed for emerging model organism genomes. *Genome Research*. 2008;**18**:188-196.

13. Tarailo-Graovac M, Chen N. Using RepeatMasker to identify repetitive elements in

genomic sequences. *Current Protocols in Bioinformatics*: 2009;**25**:4.10.1-4.10.14

14. Korf I. Gene finding in novel genomes. *BMC Bioinformatics*. 2004;**5**:1.

15. Simão FA, Waterhouse RM, Ioannidis P, Kriventseva EV, Zdobnov EM. BUSCO: assessing genome assembly and annotation completeness with single-copy orthologs. *Bioinformatics*. 2015;**31**:3210-3212.

16. Conesa A, Götz S, García-Gómez JM, Terol J, Talón M, et al. Blast2GO: a universal tool for annotation, visualization and analysis in functional genomics research. *Bioinformatics*. 2005;**21**:3674-3676.

17. Li L, Stoeckert CJ, Roos DS. OrthoMCL: identification of ortholog groups for eukaryotic genomes. *Genome Research*. 2003;**13**:2178-2189.

18. Du Z, Zhou X, Ling Y, Zhang Z, Su Z. agriGO: a GO analysis toolkit for the agricultural community. *Nucleic Acids Research*. 2010;**38**: doi:10.1093/nar/gkq310

19. Shin SC, Ahn DH, Kim SJ, Pyo CW, Lee H, et al. The genome sequence of the Antarctic bullhead notothen reveals evolutionary adaptations to a cold environment. *Genome Biology*. 2014;**15**:468.

Figure Legends

Figure 1. Photograph of Antarctic dragonfish, *P. charcoti*.

Figure 2. Estimation of the *P. charcoti* genome size based on 39-mer analysis. X-axis represents the depth (peak at 39×) and the y-axis represents the proportion. Genome size was estimated to be 805 Mb (total k-mer number/volume peak).

Figure 3. Comparative genome analyses of the *P. charcoti* genome.

A. Venn diagram of orthologous gene clusters between four arthropod lineages. B. Gene family gain-and-loss analysis. The number of gained gene families and lost gene families are indicated for each species. Time lines specify divergence times between the lineages.

Figure 1

Click here to download Figure Fig 1.jpg

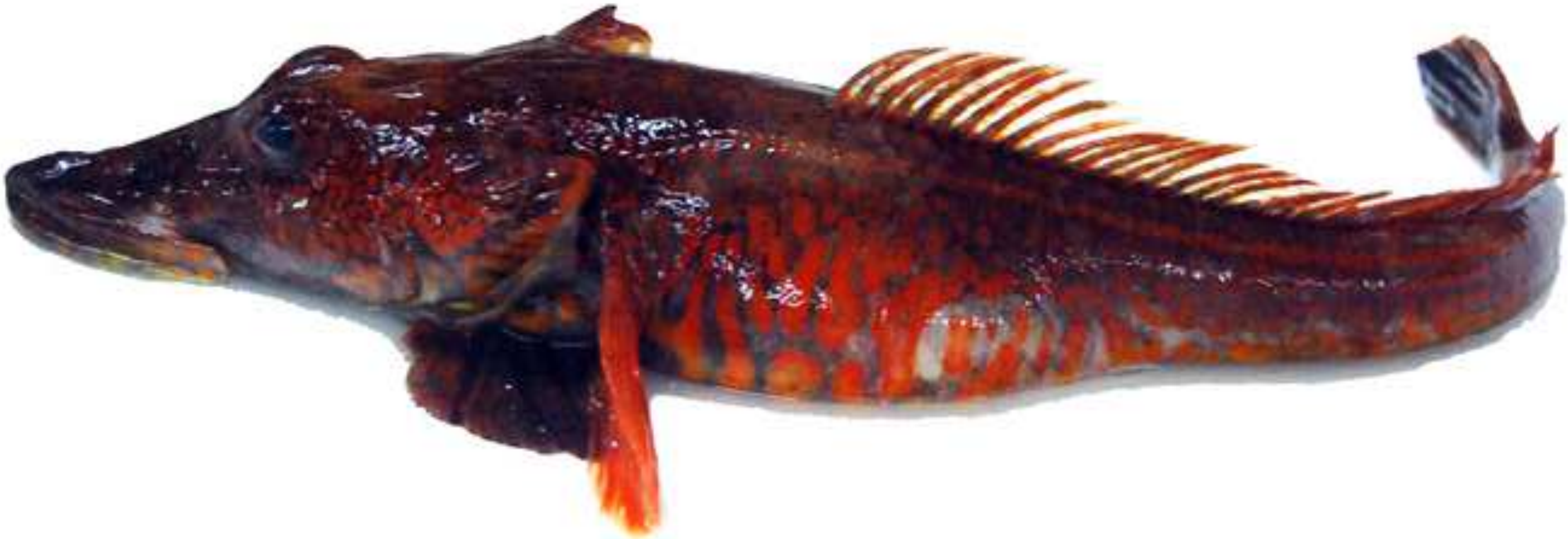

Figure 2

Click here to download Figure Fig 2.jpg

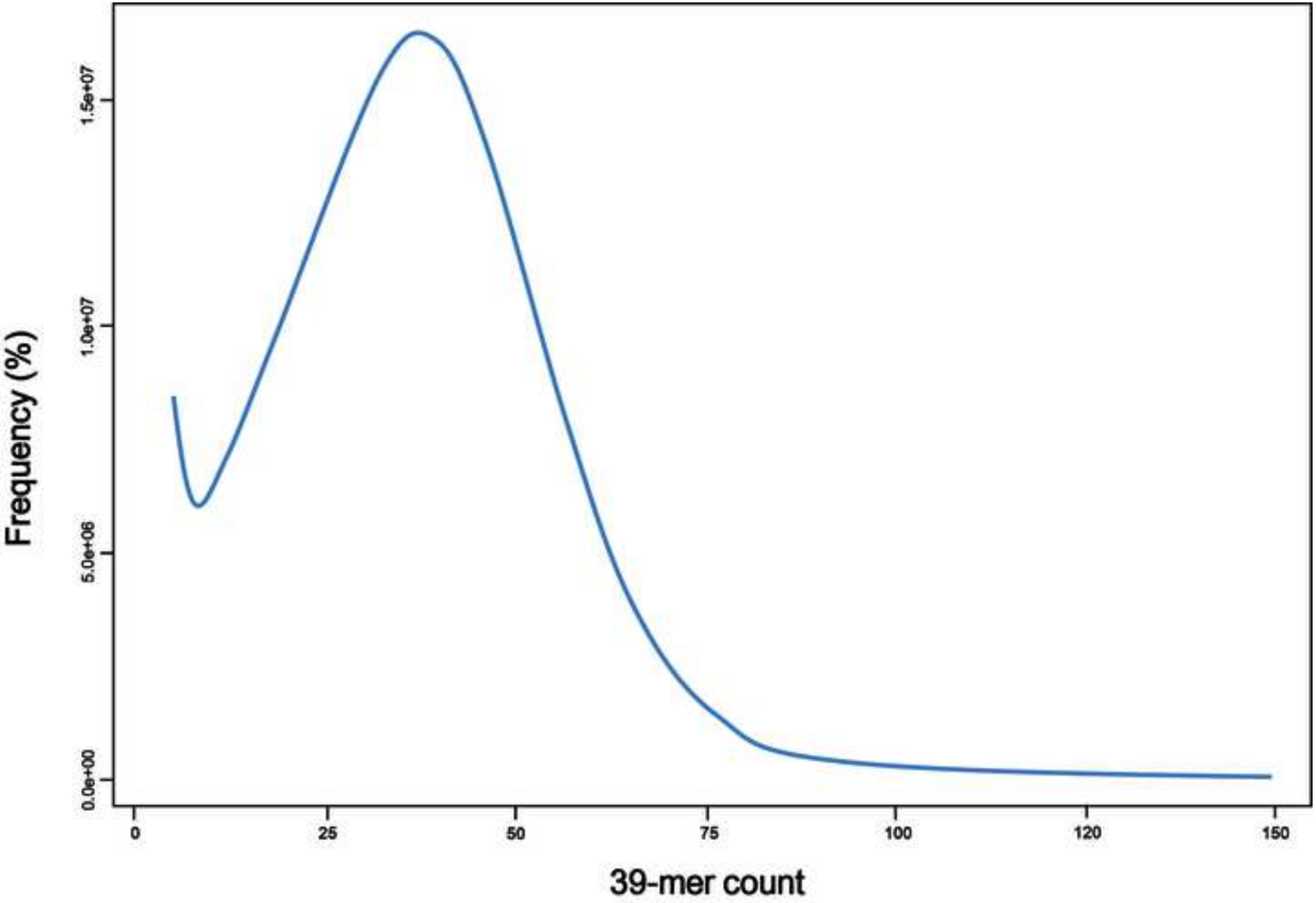

Figure 3

Click here to download Figure Fig 3.jpg

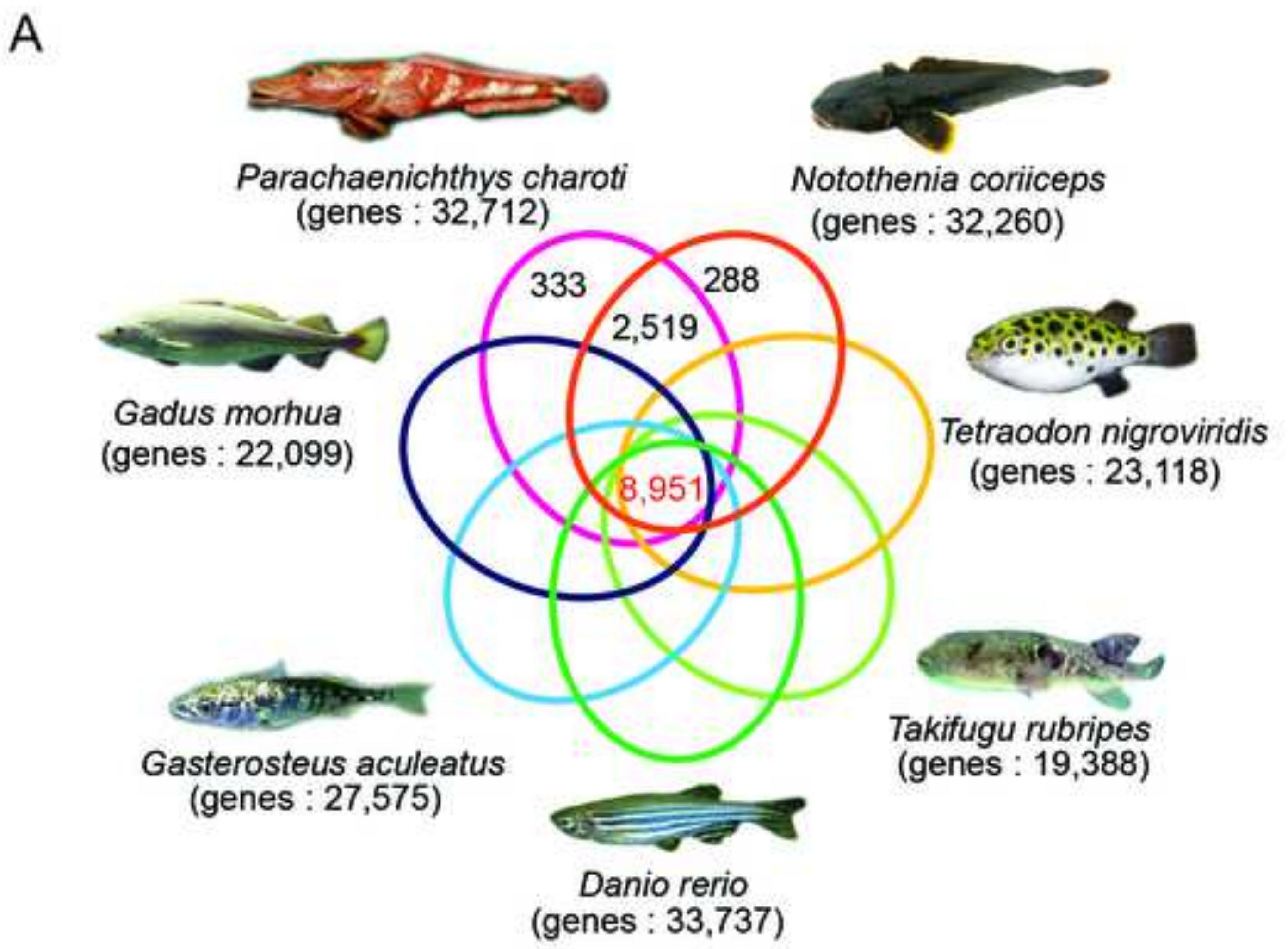

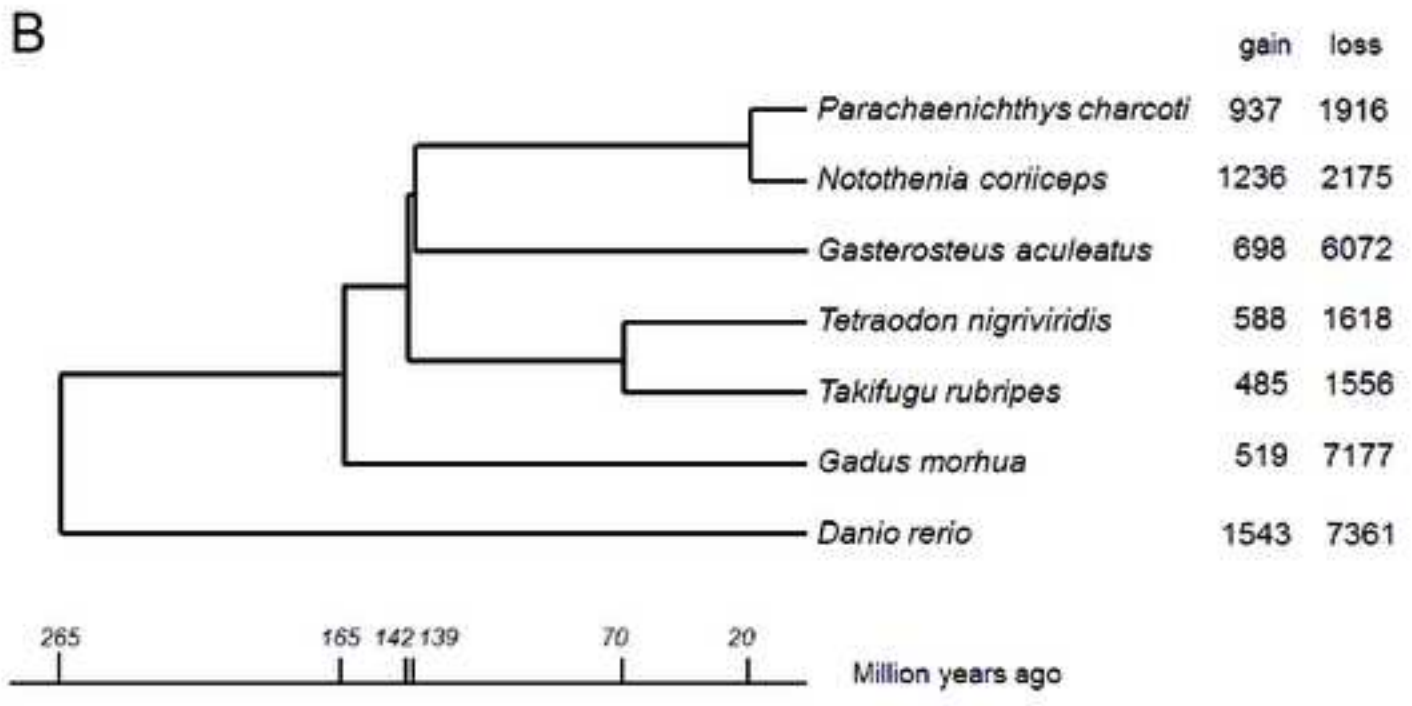

Personal Cover Click here to download Personal Cover CoverLetter-Giga.docx

Korea Polar Research Institute
(406-840) 26, Songdomirae-ro, Yeonsu-gu, Incheon, Korea
Phone : 82-32-770-8400 Fax : 82-32-770-8709

Feb 25, 2017

Dear Editor of GigaScience,

We hope you will consider our manuscript, "Draft genome of the Antarctic dragonfish, *Parachaenichthys charcoti*" as a Data note in GigaScience. All authors are aware of, and accept responsibility for, the manuscript. The accompanying manuscript is original work, not published elsewhere or under consideration for publication elsewhere.

Antarctic notothenioid teleosts have evolved to adapt to the extreme Antarctic marine environment. The fish fauna of the Southern Ocean is dominated by a single lineage belonging to the perciform suborder Notothenioidei, consisting of 132 species and 8 families. They survive in the extreme Antarctic marine environment, which includes large seasonal changes in food availability and cold ocean water. Nototheniidae is the most speciose family of the Notothenioidei, the dominant taxonomic component of Antarctic teleosts, making up 90% of the fish biomass of the continental shelf and upper slope. Parachaenichthys charcoti, the Antarctic bathydraconid dragonfish, remain almost exclusively on the inner shelves throughout their ontogeny. Several studies have investigated their ecology and ethology, but there has been no genomic study.

To identify the distinguishing characteristics of this Antarctic fish, a comprehensive genetic study is needed. We hope to provide useful data for understanding Antarctic teleost divergence and evolution. Therefore, we believe the draft *P. charcoti* genome will broaden our understanding of the evolution of Antarctic fish in their extreme environment and provide a basis information for further investigating the unusual characteristics of Antarctic fishes.

Thank you very much for your consideration of this paper,

Hyun Park,

Hyun Park, Ph. D.
Korea Polar Research Institute
26 Songdomirae-ro, Yeonsu-gu,
Incheon 21990, South Korea
Tel: +82-32-760-5570/Fax: +82-32-760-5575
e-mail: hpark@kopri.re.kr
