# Supplementary material for: Draft genome of the Antarctic dragonfish, Parachaenichthys charcoti
Source: Gigascience. 2017 Jul 24;6(8):1–6. doi: 10.1093/gigascience/gix060 (PMC5597851; doi:10.1093/gigascience/gix060)
Supplement: GIGA-D-17-00041_Revision-1.pdf [file gix060_giga-d-17-00041_revision-1.pdf]

Manuscript
Click here to download Manuscript Antarctic dragonfish-R1_ADH-fixed_02_ED.docx

Draft genome of the Antarctic dragonfish, *Parachaenichthys charcoti*

Do-Hwan Ahn[1,†], Seung Chul Shin[1,†], Bo-Mi Kim[1,†], Seunghyun Kang[1], Jin-Hyoung Kim[1], Inhye Ahn[1,2], Joonho Park[3,*], Hyun Park[1,2,*]

[1]*Unit of Polar Genomics, Korea Polar Research Institute, Incheon 21990, South Korea*

[2]*Polar Sciences, University of Science & Technology, Yuseong-gu, Daejeon 34113, South Korea*

[3]*Department of Fine Chemistry, Seoul National University of Science and Technology, Seoul 01811, South Korea*

[*]Co-corresponding authors:

Unit of Polar Genomics, Korea Polar Research Institute, Incheon 21990, South Korea; E-mail address: hpark@kopri.re.kr (H. Park)

Department of Fine Chemistry, Seoul National University of Science and Technology, Seoul 01811 South Korea; E-mail address: jhpark21@seoultech.ac.kr (J. Park)

[†]These authors contributed equally to this work.

Abstract

Background

The Antarctic bathydraconid dragonfish, *Parachaenichthys charcoti*, is an Antarctic notothenioid teleost endemic to the Southern Ocean. The Southern Ocean has cooled to −1.8C over the past 30 million years, and the seawater had retained cold temperature and isolated oceanic environment by Antarctic Circumpolar Current (ACC). Notothenioids dominate Antarctic fish, making up 90% biomass and all notothenioids have undergone molecular and ecological diversification to survive in this cold environment. Therefore, they are considered an attractive Antarctic fish model for evolutionary and ancestral genomic studies. Bathydraconidae is a speciose family of the Notothenioidei, the dominant taxonomic component of Antarctic teleosts. To understand the process of evolution of Antarctic fish, we select a typical Antarctic bathydraconid dragonfish, *P. charcoti*. Here, we have sequenced, *de novo* assembled and annotated a comprehensive genome from *P. charcoti*.

Findings

The draft genome of *P. charcoti* is 709 Mb in size. The N50 contig length is 6,145 bp and its N50 scaffold length 178,362 kb. The genome of *P. charcoti* is predicted to contain 32,712 genes, 18,455 of which have been assigned preliminary functions. A total of 8,951 orthologous groups common to seven species fish were identified, while 333 genes were identified in *P. charcoti* only; 2,519 orthologous group were also identified in both *P. charcoti* and *N. coriiceps*, another Antarctic fish. Four gene ontology (GO) terms were statistically overrepresented among the 333 genes unique to *P. charcoti*, according to GO enrichment analysis.

Conclusions

The draft *P. charcoti* genome will broaden our understanding of the evolution of Antarctic fish in their extreme environment. It will provide a basis for further investigating the unusual

characteristics of Antarctic fishes.

Keywords

Parachaenichthys charcoti, Antarctic dragonfish, Notothenioid, *De novo* genome assembly, Genome annotation.

Data description

Introduction

The fish fauna of the Southern Ocean is dominated by a single lineage belonging to the perciform suborder Notothenioidei, consisting of 132 species and 8 families. All Antarctic notothenioids have evolved to adapt to the extreme Antarctic marine environment, which includes large seasonal changes in food availability and stably cold water temperature. Notothenioids dominate Antarctic fish, making up 90% biomass and all notothenioids have undergone molecular and ecological diversification to survive in this cold environment. Therefore, they are considered an attractive Antarctic fish model for evolutionary and ancestral genomic studies. Bathydraconidae is a speciose family of the Notothenioidei, the dominant taxonomic component of Antarctic teleosts [1-4]. *Parachaenichthys charcoti*, the Antarctic bathydraconid dragonfish, was first described by Vaillant in 1906 (Notothenioidei: Bathydraconidae) (AphiaID: 234687; Fishbase ID: 7102). They are found in localities around Potter Cove, South Shetland Islands. *P. charcoti* remain almost exclusively on the inner shelves throughout their ontogeny [5]. Several studies have investigated their ecology and ethology, but there has been no genomic study [5-8]. A comprehensive genetic study is needed to identify the distinguishing characteristics of this Antarctic fish and to provide useful data for understanding Antarctic teleost divergence and evolution.

Library construction and sequencing

P. charcoti (length: ~45 cm) were collected in nets at depths of 20–30 m in Marian Cove, near King Sejong Station, on the Northern Antarctic Peninsula (62°14'S, 58°47'W) in January 2012 using the hook-and-line method (Fig. 1). High-molecular-weight genomic DNA was extracted from *P. charcoti* using the Gentra Puregene Blood Kit (Qiagen, Valencia, CA). For genomic DNA sequencing, three paired-end libraries (PE300, PE400 and PE450) were constructed from sheared genomic DNA (consisting of 300, 400 and 450 bp fragments) and subsequently prepared using standard Illumina sample preparation methods. Mate-pair libraries (MP3K, MP5K, MP8K and MP20K) were prepared for scaffolding, and sequencing was performed according to the manufacturer's instructions (consisting of 3 kb, 5 kb, 8 kb and 20 kb fragments) (Illumina, San Diego, USA).

Table 1. *P. charcoti* sequencing statistics.

Library	Mode	Insert size (bp)	Library type	Trimmed Reads	Trimmed sequence (bp)	Source
PE300	2 x 300	300	paired-end	28 776 064	4 964 428 226	Genomic DNA
PE400	2 x 300	400	paired-end	139 126 700	29 538 419 473	Genomic DNA
PE450	2 x 300	450	paired-end	85 834 292	16 644 575 781	Genomic DNA
MP3K	2 x 300	3 000	mate-pair	70 517 546	4 925 657 177	Genomic DNA
MP5K	2 x 300	5 000	mate-pair	66 623 428	4 626 486 038	Genomic DNA
MP8K	2 x 300	8 000	mate-pair	61 240 982	4 212 744 363	Genomic DNA
MP20K	2 x 300	20 000	mate-pair	86 575 644	5 387 730 972	Genomic DNA
PE500	2 x 300	500	paired-end	25 940 404	5 571 197 784	Liver RNA

Because expressed sequence tags are essential for gene annotation in draft genomes, transcriptome library was conducted using TruSeq® Sample Preparation v2 (Illumina) with

total RNA. Total RNA were extracted from liver tissue and purified using the RNeasy Mini Kit (Qiagen) with the RNase-Free DNaseI Kit (Qiagen). Extracted sample quality and concentration were determined with 2100 Bioanalyzer (Agilent Technologies, Santa Clara, CA). mRNA was isolated from 2 μg of the total RNA for double-stranded cDNA library construction with poly-A selection. For transcriptome sequencing, paired-end libraries (PE500) were constructed from sheared cDNA consisting of 500 bp fragments and subsequently prepared using standard Illumina sample preparation methods. Final transcriptome libraries length and concentration were determined with 2100 Bioanalyzer. Transcriptome libraries were sequenced using runs of 300×2 paired-end reads (Table 1).

All resulting Illumina reads were trimmed using the FASTX-Toolkit (ver. 0.0.11) (http://hannonlab.cshl.edu/fastx_toolkit) with the parameters -t 20, -l 70 and -Q 33, after which a paired sequence from the trimmed Illumina reads was selected. All sequencing processes for three paired-end libraries (genomic DNA), four mate-pair libraries (genomic DNA) and one paired-end libraries (transcriptome) were performed by Korea Polar Research Institutes (data statistics provided in Table 1).

Genome assembly

K-mer analysis was conducted using Jellyfish 2.2.5 (Jellyfish, RRID:SCR_005491) [9] to estimate the genome size from DNA paired-end libraries. The estimated genome size is 805 Mb, with the main peak observed at a coverage depth of ~39x (Fig. 2). Initial assemblies were performed using the Celera Assembler ver. 8.3 (Celera Assembler, RRID:SCR_010750) with trimmed paired-end reads [10]. For the Celera Assembler, paired-end read data were converted into FRG file format using FastqToCA, which is a utility included in the Celera Assembler. Assembly was performed on a 80-processor workstation using Intel Xeon X7460 2.66 GHz processors and 1 Tb RAM with the following parameters: overlapper = ovl,

unitigger = bogart, utgErrorRate = 0.03, utgErrorLimit = 2.5, utgGraphErrorRate = 0.030, utgGraphErrorLimit = 3.25, ovlErrorRate = 0.06, cnsErrorRate = 0.06, cgwErrorRate = 0.1, merSize = 28, doOverlapBasedTrimming = 1, merylMemory = 500000, merylThreads = 40, ovlMemory = 8 Gb, ovlThreads = 2, ovlConcurrency = 40, ovlHashBlockLength = 300000000, ovlRefBlockSize = 7630000, and ovlHashBits = 24. The initial assembly had a total size of 709 Mb, N50 contig length of 5,039 bp, and N50 scaffold length of 6,135 kb with a GC content of 40.66%. The assembled contig revealed a contig coverage of approximately 36.57x from Celera assembler. Contigs from the initial assembly were used for scaffolding using the stand-alone scaffolding tool SSPACE ver. 2.0 (SSPACE, RRID:SCR_005056) with the following parameters: -x 0, -k 3, -a 0.8, and -T 60 [11]. Trimmed mate-pair reads created using the FASTX-Toolkit were used in the scaffolding process. After scaffolding, the number of scaffolds decreased from 153,398 to 12,381, and the N50 scaffold length increased from 6,135 to 166,726 bp (Table 2). The total size of the final scaffolds (~795 Mb) was consistent with the estimated genome size (805 Mb).

Gene annotation

MAKER2 annotation pipeline (MAKER, RRID:SCR_005309) was used for genome annotation with default parameters [12]. It first identified repetitive elements using RepeatMasker ver. 3.3.0 (Repeat Masker, RRID:SCR_012954) with a *de novo* repeat library [13], which was constructed using RepeatModeler ver. 1.0.3 (RepeatModeler, RRID:SCR_015027) [14] with the Repbase library (Ver. 20140131). The SNAP gene finder [15] was selected to perform *ab initio* gene prediction from this masked genome sequence. Alignment of transcriptome assembly results using BLASTn and homologous protein information from tBLASTx were considered for gene annotation as RNA and protein evidence, respectively. Transcriptome assembly was performed by using the program CLC

Genomics Workbench 8.0 with default parameters, and sequencing reads from PE500 (Table 1) were used. Proteins from six species were used in the analysis: *Notothenia coriiceps* (NCBI reference sequence NC_015653.1) and *Danio rerio, Gasterosteus aculeatus, Takifugu rubripes*, *Tetraodon nigroviridis*, and *Gadus morhua* (all from Ensembl release 69). MAKER2 includes integration of the Annotation Edit Distance (AED) metric for controlling the quality of annotation [16]. AED values are bounded between 0 and 1, an AED value of 0 indicated that its aligned evidence and annotated gene showed an exact match. Conversely, a value of 1 indicated no evidence support. But the AED cut-off was not applied for this gene predictions. Instead, AED values were denoted in gene annotation and were considered for orthologous gene analysis and gene gain and loss.

MAKER2 was used to select and revise the final gene model based on all inputs. A total of 32,712 genes were predicted in *P. charcoti* using MAKER2 (Table 2). The annotated genes contained an average of eight exons, with an average mRNA length of 1,412 bp and CDS length of 1,291 bp. The repeat prediction from MAKER2 showed that repeat sequences accounted for 19.41% of the assembled *P. charcoti* genome.

To estimate genome assembly and annotation completeness, we performed BUSCO (Benchmarking Universal Single-Copy Orthologs) analysis (BUSCO, RRID:SCR_015008) [17], an approach used for lineage-specific profile libraries, such as those of actinopterygii, and identified 88.6% complete and 5.7% partial eukaryote orthologous gene sets in our assembly (Table 3).

To assign preliminary functions for 32,712 genes, we used Blast2GO ver. 2.6.0 (Blast2GO, RRID:SCR_005828) [18]. We classified functions for 18,455 (56.42%) predicted genes, which were annotated using BLASTp results and InterproScan (RRID:SCR_005829). Gene ontology (GO) annotation terms included “biological process” (20,126, 61.52%), “molecular function” (20,514, 62.71%), and “cellular component” (15,452, 47.23%). Enzyme

commission numbers were obtained for 3,846 proteins.

Table 2. Global statistics of the *P. charcoti* genome assembly.

		P. charcoti
Scaffold	Total scaffold length (bases)	794 596 176
	Gap size (bases)	86 840 902
	Scaffolds (n)	12 602
	N50 scaffold length (bases)	178 362
	Max scaffold length (bases)	1 318 127
Contig	Total contig length (bases)	709 540 340
	Contigs (n)	153 398
	N50 contig length (bases)	6 145
	Max contig length (bases)	65 864
Annotation	Gene Number (n)	32 712
	An average mRNA length (bases)	1 412
	An average CDS length (bases)	1 291
	An average of exons (n)	8
Repeat content (% of genome)		19.4

Table 3. Summarized benchmarks of the BUSCO (Benchmarking Universal Single-Copy Orthologs) assessment.

	**Actinopterygii (%)**
Total BUSCO groups searched	4 062*
Complete BUSCOs	88.6
Complete and single-copy	86.3
Complete and duplicated	2.3
Partial	5.7
Missing	5.7

* Number of total BUSCO groups searched

Ortholog analysis

We identified orthologous groups using OrthoMCL (ver. 2.0.5) [19], which generated a graphical representation of the sequence relationships, which were then presented in subgraphs using the Markov Clustering Algorithm based on multiple eukaryotic genomes. We used the standard parameters (percentMatchCutoff = 50 and evalueExponentCutoff = -5) and options within OrthoMCL for all steps. We used seven fish genomes for this analysis (*D. rerio, G. aculeatus, T. rubripes, T. nigroviridis, G. morhua, N. coriiceps*, and *P. charcoti*). The coding sequences of five genomes were collected from Ensembl release 69, and one coding sequence was selected among multiple proteins corresponding to one gene. We used the coding sequence from the NCBI reference sequence (NC_015653.1) of *N. coriiceps* and three groups of the coding sequence of *P. charcoti* from MAKER annotation with different AED threshold (1, 0.75, and 0.25). In case of a AED cut-off value of 1, we identified 8,951 orthologous groups common to all seven fish; 288 of 32,636 *N. coriiceps* genes and 333 of 32,712 *P. charcoti* genes were not identified in any other species, and 2,519 groups were identified only in the two Antarctic fish (Fig. 3A). When we applied a AED threshold of 0.25 against gene prediction of *P. charcoti*, 7,568 orthologous groups were identified.

Likelihood analysis of gene gain and loss

We estimated differences in the size of orthologs to identify gene families that have undergone significant size changes through evolution [20, 21]. We used the program CAFE3.0 [22] and performed analyses against three groups including the coding sequence of *P. charcoti* with different AED threshold separately. We performed phylogenetic analyses among seven representative fishes with the protein-coding gene in the orthologous groups to obtain the Newick description of a rooted and bifurcating phylogenetic tree. 8,951 orthologous gene sets were selected using the criterion of reciprocal best BLASTP hit and were aligned using PRANK (Ver. 130820) under a codon model with the “-dna -codon”

option [23], poor alignment sites were eliminated using Gblock (Ver. 0.91) under a codon model with the “-t = c” option [24]. The remaining alignment regions were concatenated, and used in the construction of the phylogenetic tree by using the neighbor-joining method in the MEGA (Ver. 6) program (MEGA, RRID:SCR_000667) [25]. The ultrametric tree of the species with branch lengths in units of time were prepared by referring TimeTree [26] for CAFE3.0 (Fig. 3B). The program was performed using $p < 0.05$, and estimated rates of birth (λ) and death (μ) were calculated using the program LambdaMu with the “-s” option. The number of gene gains and losses were calculated on each branch of the tree with the “-t” option. *P. charcoti* gained 937 and lost 1916 gene families (Fig. 3B).

The Antarctic dragonfish *P. charcoti* is a species in the sister lineage of icefishes [27-29] which is the only hemoglobinless vertebrates. The dragonfish (Bathydraconidae) and the icefish (Channichthyidae) were generally considered to be evolved from common notothenioid ancestor, which was characterized by decreased hematocrit and blood hemoglobin concentrations [30-34]. The dragonfish showed most similar patterns in these trends among red-blooded notothenioid taxa [34]. The globin complex of the dragonfish *P. charcoti* was hypothesized to be similar in length and organization to that of ancestral icefish prior to loss of functionality [35]. Along with the recently published *N. coriiceps* genome [36], the genome of *P. charcoti* will broaden our understanding of how Antarctic fish have evolved to survive in sub-zero temperatures, and might provide an important clue to understand the process of evolution to the hemoglobinless Antarctic fish and their distinct phenotypes (an increase of blood volume, low blood viscosity, large bore capillaries, increased vascularity with great capacitance, cardiomegaly, and high blood flow).

Availability of supporting data

The data for *P. charcoti* genome and transcriptome has been deposited in the Sequence Read Archive (SRA) as BioProjects PRJNA330735. Other supporting data, including annotations,

alignments and BUSCO results, are available in the *GigaScience* repository, GigaDB [37].

Competing interests

The authors declare no competing interests.

Funding

This work was supported by a grant, entitled 'Polar Genomics 101 Project (PE17080)' funded by the Korea Polar Research Institute.

Author contributions

H.P. and J.P. conceived and designed experiments and analyses. D. H. A., S.C.S., B.K., S.K., J.K., I.A. and J.P. performed experiments and conducted bioinformatics. D. H. A., S.C.S., B.K. and H.P. wrote the paper.

References

1. Eastman JT, Pratt D, Winn W. *Antarctic fish biology: evolution in a unique environment*. Academic Press San Diego; 1993.
2. Eastman JT, Clarke A. A comparison of adaptive radiations of Antarctic fish with those of nonAntarctic fish. *Fishes of Antarctica.* Springer; 1998. p. 3-26.
3. Eastman JT. Antarctic notothenioid fishes as subjects for research in evolutionary biology. *Antarctic Science.* 2000;**12**(3):276-87.
4. Eakin RR, Eastman JT, Near TJ. A new species and a molecular phylogenetic analysis of the Antarctic fish genus Pogonophryne (Notothenioidei: Artedidraconidae). *Copeia.* 2009;**4**:705-13.

5. Casaux R, Mazzotta A, Barrera-Oro E. Seasonal aspects of the biology and diet of nearshore nototheniid fish at Potter Cove, South Shetland Islands, Antarctica. *Polar Biology.* 1990;**11**(1):63-72.

6. Barrera-Oro E. The role of fish in the Antarctic marine food web: differences between inshore and offshore waters in the southern Scotia Arc and west Antarctic Peninsula. *Antarctic Science.* 2002;**14**(4):293-309.

7. Barrera-Oro E, Lagger C. Egg-guarding behaviour in the Antarctic bathydraconid dragonfish Parachaenichthys charcoti. *Polar biology.* 2010;**33**(11):1585-7.

8. Eastman JT, Sidell BD. Measurements of buoyancy for some Antarctic notothenioid fishes from the South Shetland Islands. *Polar Biology.* 2002;**25**(10):753-60.

9. Marçais G, Kingsford C. A fast, lock-free approach for efficient parallel counting of occurrences of k-mers. *Bioinformatics.* 2011;**27**(6):764-70.

10. Myers EW, Sutton GG, Delcher AL, Dew IM, Fasulo DP, Flanigan MJ, et al. A whole-genome assembly of *Drosophila*. *Science.* 2000;**287**(5461):2196-204.

11. Boetzer M, Henkel CV, Jansen HJ, Butler D, Pirovano W. Scaffolding pre-assembled contigs using SSPACE. *Bioinformatics.* 2011;**27**(4):578-9.

12. Cantarel BL, Korf I, Robb SM, Parra G, Ross E, Moore B, et al. MAKER: an easy-to-use annotation pipeline designed for emerging model organism genomes. *Genome Research.* 2008;**18**(1):188-96.

13. Tarailo-Graovac M, Chen N. Using RepeatMasker to identify repetitive elements in genomic sequences. *Current Protocols in Bioinformatics.* 2009:4-10.

14. Bao Z, Eddy SR. Automated de novo identification of repeat sequence families in sequenced genomes. *Genome Research.* 2002;**12**(8):1269-76.

15. Korf I. Gene finding in novel genomes. *BMC bioinformatics.* 2004;**5**(1):59.

16. Holt C, Yandell M. MAKER2: an annotation pipeline and genome-database management

tool for second-generation genome projects. *BMC bioinformatics.* 2011;**12**(1):491.

17. Simão FA, Waterhouse RM, Ioannidis P, Kriventseva EV, Zdobnov EM. BUSCO: assessing genome assembly and annotation completeness with single-copy orthologs. *Bioinformatics.* 2015:btv351.

18. Conesa A, Gotz S, Garcia-Gomez JM, Terol J, Talon M, Robles M. Blast2GO: a universal tool for annotation, visualization and analysis in functional genomics research. *Bioinformatics.* 2005;**21**(18):3674-6.

19. Li L, Stoeckert CJ, Jr., Roos DS. OrthoMCL: identification of ortholog groups for eukaryotic genomes. *Genome Research.* 2003;**13**(9):2178-89.

20. Hahn MW, De Bie T, Stajich JE, Nguyen C, Cristianini N. Estimating the tempo and mode of gene family evolution from comparative genomic data. *Genome Research.* 2005;**15**(8):1153-60.

21. Hahn MW, Han MV, Han S-G. Gene family evolution across 12 *Drosophila* genomes. *PLoS Genetics.* 2007;**3**(11):e197.

22. De Bie T, Cristianini N, Demuth JP, Hahn MW. CAFE: a computational tool for the study of gene family evolution. *Bioinformatics.* 2006;**22**(10):1269-71.

23. Loytynoja A, Goldman N. An algorithm for progressive multiple alignment of sequences with insertions. *Proceedings of the National Academy of Sciences of the United States of America.* 2005;**102**(30):10557-62.

24. Castresana J. Selection of conserved blocks from multiple alignments for their use in phylogenetic analysis. *Molecular Biology and Evolution.* 2000;**17**(4):540-52.

25. Tamura K, Stecher G, Peterson D, Filipski A, Kumar S. MEGA6: molecular evolutionary genetics analysis version 6.0. *Molecular Biology and Evolution.* 2013;**30**(12):2725-9.

26. Hedges SB, Dudley J, Kumar S. TimeTree: a public knowledge-base of divergence times among organisms. *Bioinformatics.* 2006;**22**(23):2971-2.

27. Balushkin A, Voskoboynikova O. Systematics and phylogeny of antarctic dragonfishes (Bathydraconidae, Notothenioidei, Perciformes). *Journal of ichthyology.* 1995;**35**(5):89-104.

28. Derome N, Chen W-J, Dettaı A, Bonillo C, Lecointre G. Phylogeny of Antarctic dragonfishes (Bathydraconidae, Notothenioidei, Teleostei) and related families based on their anatomy and two mitochondrial genes. *Molecular phylogenetics and evolution.* 2002;**24**(1):139-52.

29. Near TJ, Parker SK, Detrich HW. A genomic fossil reveals key steps in hemoglobin loss by the antarctic icefishes. *Molecular biology and evolution.* 2006;**23**(11):2008-16.

30. Bargelloni L, Marcato S, Patarnello T. Antarctic fish hemoglobins: evidence for adaptive evolution at subzero temperature. *Proceedings of the National Academy of Sciences.* 1998;**95**(15):8670-5.

31. Beers JM, Borley KA, Sidell BD. Relationship among circulating hemoglobin, nitric oxide synthase activities and angiogenic poise in red-and white-blooded Antarctic notothenioid fishes. *Comparative Biochemistry and Physiology Part A: Molecular & Integrative Physiology.* 2010;**156**(4):422-9.

32. D'Avino R, Di Prisco G. Antarctic fish hemoglobin: an outline of the molecular structure and oxygen binding properties—I. Molecular structure. *Comparative Biochemistry and Physiology Part B: Comparative Biochemistry.* 1988;**90**(3):579-84.

33. Di Prisco G. Molecular adaptations of Antarctic fish hemoglobins. *Fishes of Antarctica*: Springer; 1998. p. 339-53.

34. Kunzmann A, Caruso C, di Prisco G. Haematological studies on a high-Antarctic fish: Bathydraco marri Norman. *Journal of experimental marine biology and ecology.* 1991;**152**(2):243-55.

35. Lau YT, Parker SK, Near TJ, Detrich HW, 3rd. Evolution and function of the globin

intergenic regulatory regions of the antarctic dragonfishes (Notothenioidei: Bathydraconidae). *Molecular biology and evolution.* 2012;**29**(3):1071-80.

36. Shin SC, Ahn DH, Kim SJ, Pyo CW, Lee H, Kim MK, et al. The genome sequence of the Antarctic bullhead notothen reveals evolutionary adaptations to a cold environment. *Genome Biology.* 2014;**15**(9):468.

37. Ahn DH, Shin SC, Kim BM, Kang S, Kim JH, Ahn I, Park J, Park H. Supporting data for "Draft genome of the Antarctic dragonfish, *Parachaenichthys charcoti*". GigaScience Database. 2017. http://dx.doi.org/10.5524/100321

Figure Legends

Figure 1. Photograph of Antarctic dragonfish, *P. charcoti.*

Figure 2. Estimation of the *P. charcoti* genome size based on 39-mer analysis. X-axis represents the depth (peak at 39×) and the y-axis represents the proportion. Genome size was estimated to be 805 Mb (total k-mer number/volume peak).

Figure 3. Comparative genome analyses of the *P. charcoti* genome.

A. Venn diagram of orthologous gene clusters between four arthropod lineages. B. Gene family gain-and-loss analysis. The number of gained gene families and lost gene families are indicated for each species. Time lines specify divergence times between the lineages.

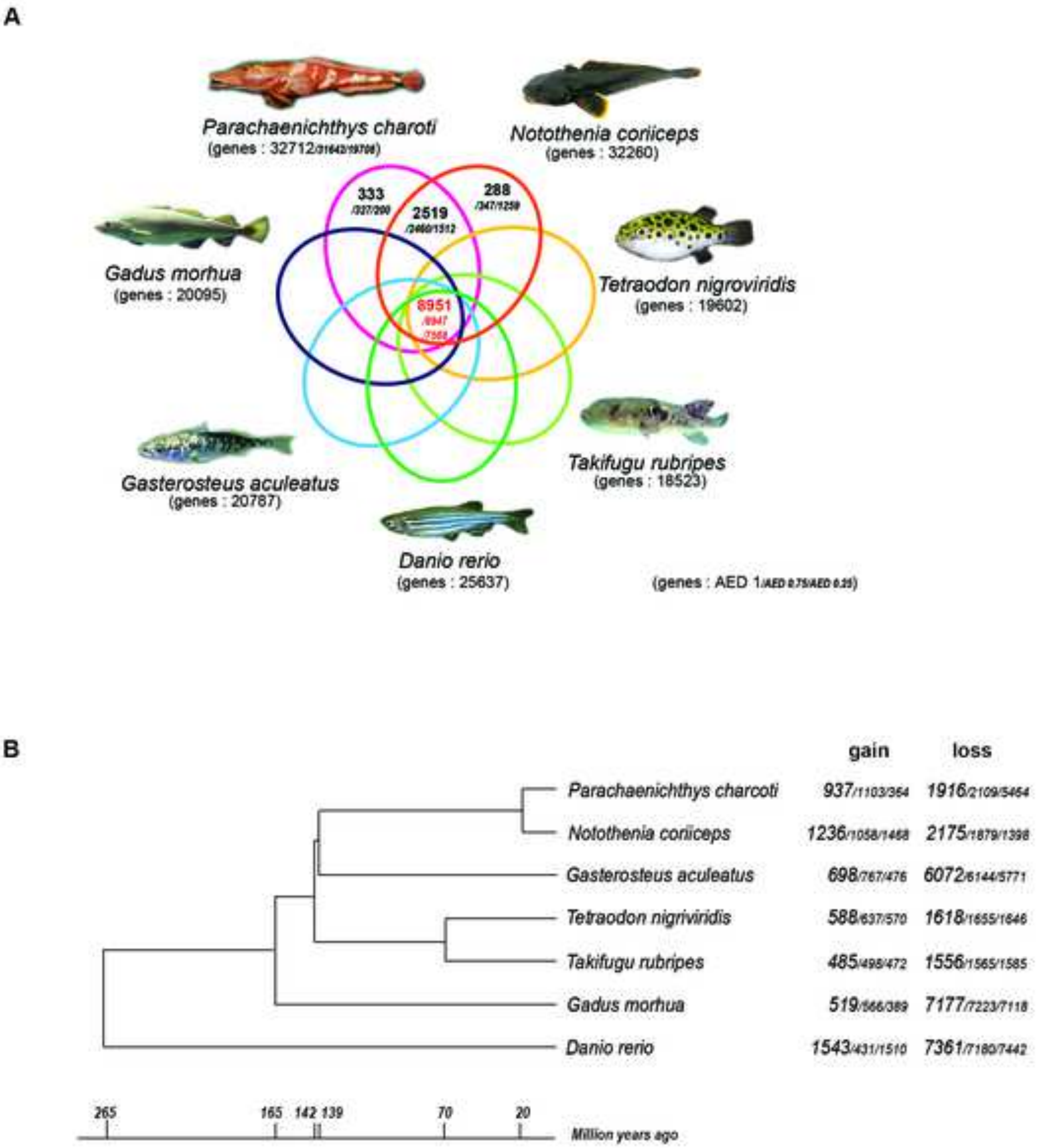
A
Parachaenichthys charoti
(genes : 32712)
Notothenia coriiceps
(genes : 32260)
Gadus morhua
(genes : 20095)
Tetraodon nigroviridis
(genes : 19602)
Gasterosteus aculeatus
(genes : 20787)
Takifugu rubripes
(genes : 18523)
Danio rerio
(genes : 25637)
333
2519
288
8951
(genes : AED 1
B
gain
loss
Parachaenichthys charcoti
937/1103/364
1916/2109/5464
Notothenia coriiceps
1236/1058/1468
2175/1879/1398
Gasterosteus aculeatus
698/767/476
6072/6144/5771
Tetraodon nigriviridis
588/637/570
1618/1655/1646
Takifugu rubripes
485/498/472
1556/1565/1585
Gadus morhua
519/566/389
7177/7223/7118
Danio rerio
1543/431/1510
7361/7180/7442
265
165
142
139
70
20
Million years ago

response to reviewer, rebuttal letter
Click here to download Personal Cover revision_R1_ADH-fixed_02_Plain text.docx

Reviewer reports:

Reviewer #1: The manuscript "Draft genome of the Antarctic dragonfish, Parachaenichthys charcoti" is another important contribution to the scientific community working on comparative teleost genomics. As with similar studies, this manuscript appears to be submitted with the purpose of releasing this valuable dataset to the public, and holds no claim to solve any specific scientific question, but rather put up possibilities for the future use of this dataset.

Major comments

The authors have made a good attempt to conduct thorough sequencing of the Antarctic dragonfish genome, using several paired-end and mate-pair libraries. However, the results, and especially the N50 contig statistic is far below what this reviewer would expected using Celera Assembler (CA) with the sequencing data presented. This reviewer is curious to why this specific sequencing method was applied (i.e three very similar libraries for PE sequencing and 2x300bp).

Author's response:
We planned to assemble sequencing reads into contigs using various assembler programs from the same sequencing libraries: Abyss, ALLPATHS-LG, SOAPdenovo, and Celera assembler were used as assemblers in this study. In case of ALLPATHS-LG, paired-end reads should be merged into single read to assemble using higher k-mer. So, we designed the length of libraries to be shorter than 500 bases. The longer reads were known to be favorable in assemblies using *de Bruijn* graph methods and overlap-layout-consensus methods. So sequencing libraries were sequenced with 2x300bp mode using Illumine MiSeq.

For all the paired-end libraries the inserts are shorter than the sequencing output, which appears to be quite wasted as the trimmed reads are only 173-212bp on average for these libraries. Would it not have been better to have libraries with an insert size around 700-800bp? This would surely span many more of the repetitive sequences now causing gaps and low continuity.Also, as trimming is part of the CA pipeline, why trim the reads prior to running CA? Additionally, FLASH should have been applied to merge overlapping reads from the paired-end sequencing libraries prior to assembly.

Author's response:
As reviewer's comments, if libraries with an insert size around 700-800bp were used, the assembly statistics would be better from Celera assembler. But we were greedy to create libraries that meets all conditions in assemblers and construct libraries with the an insert size up to 500bp to be merged.

In Celera assembler, windows below average quality value of 12 are trimmed as default. We wanted to use only sequencing reads with high quality in assemblies with Celera assembler and other assemblers using *de Bruijn* graph, and trimmed the bases with a low quality score < 20 from 3'-end of reads. After that, the reads shorter than 70 in length were also discarded, and the resulting high quality reads were used in all assemblies. The use of FLASH is a good suggestion and we will apply it later to improve the genome assembly.

The authors have also made a fair attempt to annotate this P. charcoti draft genome using the MAKER pipeline, and I'm happy to see that effort has been put into RNA sequencing to improve this analysis. However, some shortcuts have been taken in regard to how the annotation was performed. For instance, it is now standard procedure to produce a species specific repeat library, using RepeatModler to aid in the annotation. This was not done. The authors also fail to inform which library that was used for identifying repetetive elements with RepeatMasker. It is also customary to include SNAP, AUGUSTUS and GENEFINDER runs as part of the MAKER pipeline to improve gene prediction. This reviewer cannot see that this has been included in the annotation pipeline, which might explain why the number of predicted genes is so high. I'm also missing information regarding which AED cut-off that was used for the final gene predictions.

Author's response:

We used *de novo* repeat library to identify repetitive elements using RepeatMasker, and the *de novo* repeat library were produced using repeatModeler with the Repbase library (Ver. 20140131). We also selected the SNAP in MAKER annotation pipeline. Because predicted genes with AED score less than 0.75 were about 3%, we used 1 as AED cut-off values for the final gene predictions. The number of genes with AED value below 0.75 and below 0.25 was 31,642 and 19,708, respectively. We remained the gene with high AED value for manual review, added AED value into the file called "Blast2Go_annotation_with_AED.tab" in GigaDB, and we changed manuscript as follows:

"MAKER2 annotation pipeline was used for genome annotation with default parameters [12]. It first identified repetitive elements using RepeatMasker (ver. 3.3.0) with a *de novo* repeat library [13], which was constructed using RepeatModeler (Ver. 1.0.3) [14] with the Repbase library (Ver. 20140131). The SNAP gene finder [15] was selected to perform ab initio gene prediction from this masked genome sequence. Alignment of transcriptome assembly results using BLASTn and homologous protein information from tBLASTx were considered for gene annotation as RNA and protein evidence, respectively. Transcriptome assembly was performed by using the program CLC Genomics Workbench 8.0 with default parameters, and sequencing reads from PE500 (Table 1) were used. Proteins from six species were used in the analysis: *Notothenia coriiceps* (NCBI reference sequence NC_015653.1) and *Danio rerio*, *Gasterosteus aculeatus*, *Takifugu rubripes*, *Tetraodon nigroviridis*, and *Gadus morhua* (all

from Ensembl release 69). MAKER2 include integration of the Annotation Edit Distance (AED) metric for controlling the quality of annotation [16]. AED values are bounded between 0 and 1, an AED value of 0 indicated that its aligned evidence and annotated gene showed an exact match, Conversely, a value of 1 indicated no evidence support. But the AED cut-off was not applied for this gene predictions. Instead, AED values were denoted in gene annotation and were considered for orthologous gene analysis and gene gain and loss.”

The authors have further investigated the gene space completeness using BUSCO, which is good. However, there is reasons to belive that the gene sets reported are not up to date, especially since there is now a Actinopterygii specific gene set available (http://busco.ezlab.org/frame_meta.html). This should be quick to run and the results can easily be implemented in Table 3.

Author’s response:
We did re-run BUSCO analysis to the Actinopterygii DB, and changed Table 3 with new results as reviewer’s comments.

In an attempt to conduct comparative genomics, the authors have grouped orthologous genes from several species into orthologous groups using OrthoMCL. This is an OK starting point for a comparative analysis, however, their analysis is based on unfiltered data for the ENSEMBLE (which is know to include thousands of duplicates and Gene ID's without any sequence data available). For instance, would 24,460 genes be a much more adequate dataset to use for the zebrafish. It also included all of the 32,712 P. charcoti gene predictions, which leads me to belive that most of the 333 othologous groups (according to Figure 3a, yet referred to as 333 genes" in the text) contain false positives and/or repeats. Based on these results, the authors also produce a "gain-and-loss" figure for the investigated species, yet there is no mentioning on how this analysis was performed.

Author’s response:
We filtered the data from the ENSEMBLE, we selected one gene among transcript variants, and discarded the gene without any sequence data available. Then, we have grouped orthologous genes with filtered data into orthologous groups. 25,637 zebra fish gene were used in this analysis, and the number of filtered genes for the ENSEMBLE were indicated in Figure 3A. We did not filtered the 32,712 *P. charcoti* gene predictions completely. Instead, we performed the analysis with the genes corresponding to the three conditions (AED cut-off;1, 0.75, 0.25) and the results corresponding to each case were shown in Figure 3A and 3B. The method producing a “gain-and-loss” was added to manuscript. We also changed the manuscripts as follow:

“*Likelihood analysis of gene gain and loss*

We estimated differences in the size of orthologs to identify gene families that have undergone significant size changes through evolution [20, 21]. We used the program CAFE3.0 [22] and performed analyses against three groups including the coding sequence of P. charcoti with different AED threshold separately. We performed phylogenetic analyses among seven representative fishes with the protein-coding gene in the orthologous groups to obtain the Newick description of a rooted and bifurcating phylogenetic tree. 8,951 orthologous gene sets were selected using the criterion of reciprocal best BLASTP hit and were aligned using PRANK (Ver. 130820) under a codon model with the "-dna -codon" option [23], poor alignment sites were eliminated using Gblock (Ver. 0.91) under a codon model with the "-t = c" option [24]. The remaining alignment regions were concatenated, and used in the construction of the phylogenetic tree by using the neighbor-joining method {Saitou, 1987 #51} in the MEGA (Ver. 6) program [25]. The ultrametric tree of the species with branch lengths in units of time were prepared by referring TimeTree [26] for CAFE3.0 (Figure 3B). The program was performed using $p < 0.05$, and estimated rates of birth (λ) and death (μ) were calculated using the program LambdaMu with the "-s" option. The number of gene gains and losses were calculated on each branch of the tree with the "-t" option. *P. charcoti* gained 937 and lost 1916 gene families (Figure 3B)."

Finally, the authors also present analyses based on (crude) Gene Ontology analyses which offer little scientific value. The entire paragraph on GO enrichment testing (including the results) it not very interesting. So, unless there is any biological meaning applied to the genes or pathways identified, this could/should be removed.

Author's response:
We removed the paragraph and tables for the gene ontology analyses according to reviewer's suggestion.

Minor comments

i) Please use an appropriate "thousands seperator" for all values across the manuscript

Content were corrected: content of Table1-3.

ii) Please make sure that the genus name is not spelled out several times.

Content were corrected: *Parachaenichthys charcoti* to *P. charcoti*

iii) Excange "illumine" for "Illumina" prior to Table 1

Modification of content: "illumine" to (Illumina, San Diego, USA)

Reviewer #2: Review of Manuscript GIGA-D-17-00041

Overview

Hyun Park's group present the first genome sequence for Parachaenichthys charcoti, a member of the bathydraconid (Antarctic dragonfish) clade of the notothenioid group of Antarctic teleosts. This is the second notothenioid genome to be made publically available, following the publication of the Antarctic bullhead Notothenia coriiceps (Shin SC et al. Genome Biology. 2014;15:468). As a fish biologist interested in physiological evolution, the availability of multiple notothenioid genomes presents a great opportunity for deciphering the genomic basis of adaptive/non-adaptive changes made possible by the extre me cold environment and unusual evolutionary history linked to the notothenioid radiation. As a resource, the P. charcoti genome will be used for comparative analyses with N. coriiceps and other teleost genomes. I am particularly excited about the eventual publication of a genome for an Antarctic icefish species (Channichthyidae), for which the most extreme physiological traits linked to cold conditions are observed (e.g. total loss of haemoglobin). The genomes of N. corriceps and P. charcoti will be crucial for such comparative analyses. It is important to note that the Antarctic dragonfishes and Notothenia lineages are relatively distant, so the availability of both genome sequences allows both shared-ancestral and lineage-specific changes or adaptations to be disentangled. Moreover, these genomes are generally important in the context of understanding the physiological capacity of notothenioids - key to the overall fauna of Antarctica - to respond to contemporary changes in climate. The manuscript is generally well written.

Thus, overall, I support the publication of this Data Note in GigaScience and I think the paper will encourage the uptake of the P. charcoti genome for a range of physiological and evolutionary questions. The data provided by the authors is generally comprehensive and relevant. I offer a number of comments/suggestions, aiming to either increase the clarity surrounding the manuscript's organization and the data and its applications, or requesting more details on aspects of the methodology. I split my comments into general suggestions and a larger set of minor points, the latter linked to particular text in the paper.

General suggestions

1. The authors might consider adding an informative heading to the first paragraph of the Data Description section, such as 'Context' or "Background". This would increase the clarity of the manuscript's organization.

Author’s response:

We added “*Introduction*” as an informative heading.

2. I suggest that authors include an additional dedicated section at the end of the manuscript along the lines of the "Re-use potential" subheading suggested in the Journal guidelines. At the moment, the paper does not do a very proficient job in helping the reader envisage specific uses for the Data Set presented. Hence, in current form, the wider interest of the data set is not fully justified. I would like to see elaboration of the author's stance concerning data re-use, which I feel is necessary to meet the Journal's aim to "contextualize exceptional datasets to encourage reuse". This could provide more context in light of the findings of Shin et al. 2014 (e.g. the new P. charcoti genome will allow questions such as, which genomic traits are ancestral to all notothenioids? Which are lineage-specific? Which evolved by convergence? etc.), or give more context on interesting physiological traits observed in notothenioids for which researchers are seeking to clarify the underlying genomic basis.

Author’s response:

We added an additional section at the end of the manuscript to satisfy for “Re-use potential” as follow: “The Antarctic dragonfish *P. charcoti* is a species in the sister lineage of icefishes [27-29] which is the only hemoglobinless vertebrates. The dragonfish (Bathydraconidae) and the icefish (Channichthyidae) were generally considered to be evolved from common notothenioid ancestor, which was characterized by decreased hematocrit and blood hemoglobin concentrations [30-34]. The dragonfish showed most similar patterns in these trends among red-blooded notothenioid taxa [34]. The globin complex of the dragonfish *P. charcoti* was hypothesized to be similar in length and organization to that of ancestral icefish prior to loss of functionality [35]. Along with the recently published *N. coriiceps* genome [36], the genome of *P. charcoti* will broaden our understanding of how Antarctic fish have evolved to survive in sub-zero temperatures, and might provide an important clue to understand the process of evolution to the hemoglobinless Antarctic fish and their distinct phenotypes (an increase of blood volume, low blood viscosity, large bore capillaries, increased vascularity with great capacitance, cardiomegaly, and high blood flow). “

3. I find the GO analyses to have tangential relevance as a dataset of meaningful future use, unless it is dissected considerably more than presented within this Data Note, where it appears much as a 'bolt-on'. The biological meaning of data presented in Table 4 (overrepresented GO terms in P. charcoti) does not add much insight to fuel on-going research. The data in Table 5 may be misleading in terms of its potential meaning for notothenioid-specific evolution, since the gene list was defined on the basis of comparing two notothenioids with stickleback as the next nearest phylogenetic lineage. As tens of millions of years separate notothenioids and stickleback, it is impossible to say the genes are restricted

to notothenioids. This is a minor point, but for me, the paper would be clearer without the GO analyses.

Author’s response:

We removed the paragraph and tables for the gene ontology analyses according to reviewer’s suggestion.

4. The authors should use species abbreviations consistently throughout the manuscript, which is not the case currently.

Author’s response:

We corrected species abbreviation throughout the manuscript.

5. The authors used Celera to assemble the paired end MiSeq reads. As this is an OLC assembler, I would not have naturally considered this to be an optimal approach using relatively short read lengths provided by MiSeq. However, the authors provide evidence that a reasonable draft genome and annotation was nonetheless generated. I am intrigued, in a comparative sense, to know how the same data would have performed using the best-performing assemblers built on the de Bruijn Graph approach. Did the authors attempt any such assemblies, and if so, why did they eventually choose to go with the Celera assembly? To clarify, I am not requesting this as a necessary revision, though if the authors had some available data, I feel it would be of wider interest to contrast the performance of different assemblers.

Author’s response:

We assembled sequencing reads into contigs using various assembler from the same sequencing libraries: Abyss, ALLPATHS-LG, SOAPdenovo, and Celera assembler. The assembly statistics from Celera assembler were best among assemblers.

	P. charcoti	**CA 8.3**	**Abyss 2.0.2**	**SOAPdenovo2**	**Allpath-LG**
Scaffold	Total scaffold length (bases)	794 596 176	1 460 857 469	1,130,003,516	685,815,544
	Gap size (bases)	86 840 902	385 080 136	529,475,795	172,038,706
	Scaffolds (n)	12 602	5 921 399	785,432	29,613
	N50 scaffold length (bases)	178 362	10 786	50,086	74,560
	Max scaffold length (bases)	1 318 127	993 314	691,673	716,090
Contig	Total contig length (bases)	709,540,340	1,076,189,796	607,268,662	529,876,330
	Contigs (n)	153,398	6,198,487	2,431,352	139,649
	N50 contig length (bases)	6,145	279	313	6,067
	Max contig length (bases)	65,864	32,177	3,493	67,562

Gene Number (n)		32,712			
Repeat content (% of genome)		19.4			
BUSCO	Complete BUSCOs (%)	88.6	75.9	78.9	65
	Complete and single-copy BUSCOs (%)	86.3	74	76.9	64
	Complete and duplicated BUSCOs (%)	2.3	1.9	2	2
	Fragmented BUSCOs (%)	5.7	13.3	9.4	18
	Missing BUSCOs (%)	5.7	10.8	11.7	17
	Total BUSCO groups searched (n)	4 584*			

* Total number of Actinopterygii database

Specific minor points

1. Abstract: "… and P. charcoti has undergone molecular and ecological diversification to survive in this cold environment". The wording here can be misconstrued, as the same statement is true for the wider notothenioid lineage. Better to write "… and all notothenioids have undergone molecular and ecological diversification to survive in this cold environment.

Modification of content:

Notothenioids dominate Antarctic fish, making up 90% biomass and all notothenioids have undergone molecular and ecological diversification to survive in this cold environment.

2. "However, little is known about the biology of this species, except that globin intergenic regulatory regions play a role in its low levels of alpha-globin expression". I found this sentence a little disappointing as an upfront motivation for the Data. I feel the abstract could more strongly communicate the importance of the target species for our comparative understanding of evolution in Antarctic fish. Which genomic traits are ancestral to notothenioids, which are lineage-specific, which evolved by convergence, etc.? I suspect these are the motivating questions and in my opinion, the paper would be stronger if this came through more strongly generally, including the abstract.

Modification of content:

Therefore, they (notothenioids) are considered an attractive Antarctic fish model for evolutionary and ancestral genomic studies. Bathydraconidae is a speciose family of the Notothenioidei, the dominant taxonomic component of Antarctic teleosts. To understand the process of evolution of Antarctic fish, we select a typical Antarctic bathydraconid dragonfish, *P. charcoti*.

3. Keywords: the authors might consider elaborating this list, for example to include mention of a genome assembly. Currently the keyword list could be linked to almost any field where Antarctic fish are studied, so it should better represent a genome biology paper.

Modification of content:

Keywords: *Parachaenichthys charcoti*, Antarctic dragonfish, Notothenioid, *De novo* genome assembly, Genome annotation.

4. Data description paragraph 1: "Antarctic notothenioid teleosts have evolved to adapt to the extreme Antarctic marine environment. The fish fauna of the Southern Ocean is dominated by a single lineage belonging to the perciform suborder Notothenioidei, consisting of 132 species and 8 families. They survive in the extreme Antarctic marine environment, which includes large seasonal changes in food availability and cold ocean water.

These first few sentences have an issue with the flow of information, which jumps about abruptly, as if thrown together. Consider a reformulation: "The fish fauna of the Southern Ocean is dominated by a single lineage belonging to the perciform suborder Notothenioidei, consisting of 132 species and 8 families. All Antarctic notothenioids have evolved to adapt to the extreme Antarctic marine environment, which includes large seasonal changes in food availability and stably cold water temperature."

Modification of content:

The fish fauna of the Southern Ocean is dominated by a single lineage belonging to the perciform suborder Notothenioidei, consisting of 132 species and 8 families. All Antarctic notothenioids have evolved to adapt to the extreme Antarctic marine environment, which includes large seasonal changes in food availability and stably cold water temperature.

5. Data description paragraph 1: "Nototheniidae is the most speciose family of the Notothenioidei, the dominant taxonomic component of Antarctic teleosts, making up 90% of the fish biomass of the continental shelf and upper slope [1-4]. Parachaenichthys charcoti, the Antarctic bathydraconid dragonfish, was first described by Vaillant in 1906".

I find the construction of these sentences to be unusual - when first reading the information, the implication I got was that P. charcoti is a member of Nototheniidae, which is not the case. Can the authors please address the construction of the text to improve the clarity of the information?

Modification of content:

Notothenioids dominate Antarctic fish, making up 90% biomass and all notothenioids have undergone molecular and ecological diversification to survive in this cold environment. Therefore, they are considered an attractive Antarctic fish model for evolutionary and ancestral genomic studies. Bathydraconidae is a speciose family of the Notothenioidei, the dominant taxonomic component of Antarctic teleosts [1-4]. *Parachaenichthys charcoti*, the Antarctic bathydraconid dragonfish, was first described by Vaillant in 1906 (Notothenioidei: Bathydraconidae) (AphiaID: 234687; Fishbase ID: 7102) .

6. Page 4, "All sequencing …. (Table 1)", would read more clearly as "All sequencing …. (data statistics provided in Table 1)". In the current form, the table citation is not clearly linked to the provided text about 'sequencing processes'.

Content were corrected:

For genomic DNA sequencing, three paired-end libraries (PE300, PE400 and PE450) were constructed from sheared genomic DNA (consisting of 300, 400 and 450 bp fragments) and subsequently prepared using standard Illumina sample preparation methods. Mate-pair libraries (MP3K, MP5K, MP8K and MP20K) were prepared for scaffolding, and sequencing was performed according to the manufacturer's instructions (consisting of 3 kb, 5 kb, 8 kb and 20 kb fragments) (Illumina, San Diego, USA).

Because expressed sequence tags are essential for gene annotation in draft genomes, transcriptome library was conducted using TruSeq® Sample Preparation v2 (Illumina) with total RNA. Total RNA were extracted from liver tissue and purified using the RNeasy Mini Kit (Qiagen) with the RNase-Free DNaseI Kit (Qiagen). Extracted sample quality and concentration were determined with 2100 Bioanalyzer (Agilent Technologies, Santa Clara, CA). mRNA was isolated from 2 μg of the total RNA for double-stranded cDNA library construction with poly-A selection. For transcriptome sequencing, paired-end libraries (PE500) were constructed from sheared cDNA consisting of 500 bp fragments and subsequently prepared using standard Illumina sample preparation methods. Final transcriptome libraries length and concentration were determined with 2100 Bioanalyzer. Transcriptome libraries were sequenced using runs of 300×2 paired-end reads (Table 1).

All resulting Illumina reads were trimmed using the FASTX-Toolkit (ver. 0.0.11) (http://hannonlab.cshl.edu/fastx_toolkit) with the parameters -t 20, -l 70 and -Q 33, after which a paired sequence from the trimmed Illumina reads was selected. All sequencing processes for three paired-end libraries (genomic DNA), four mate-pair libraries (genomic DNA) and one paired-end libraries (transcriptome) were performed by Korea Polar Research Institutes (data statistics provided in Table 1).

7. Page 4, "illumine, Carlsbad, USA". Please correct the typo.

Content were corrected:
"illumine, Carlsbad, USA" to “Illumina, San Diego, USA”

8. Page 4, "Finally, paired-end trimmed reads data with 73-fold coverage were obtained (Table 1). How was the fold-coverage estimated in this case? Also, why present coverage just for the paired-end libraries and not the mate pair libraries?

Author’s response:
We divided the sum of paired-end trimmed sequence by the predicted genome size to calculate the fold-coverage. Because the mate-pair libraries were used only in scaffolding, we did not considered it as coverage. But this sentence was not informative. So we deleted this sentence.

9. Page 5: "The assembled contig revealed a contig coverage of approximately 36.57x". By what approach was this assessed?

Author’s response:
A contig coverage were calculated by Celera assembler, so we added “in Celera assembler” at the end of the sentence as follow:
“The assembled contig revealed a contig coverage of approximately 36.57x from Celera assembler.”

10. Page 5: Why were the selected parameters in Celera selected? Are these simply generally optimized default parameters?

Author’s response:
We had tried some optimized Celera assembler parameters, but default option generated best result, although some parameter was optimized for our computer power. Our experience was identical to other genome cases.

11. Page 5: "Contigs from the initial assembly were used for scaffolding using the stand-alone scaffolding tool SSPACE (ver. 2.0) [11]. Trimmed mate-pair reads created using the FASTX-Toolkit were used in the scaffolding process".
Can the authors please provide enough information on the SSPACE parameters employed to allow the reader to repeat the analysis?

We added the parameters at the end of the sentence as follow:
“Contigs from the initial assembly were used for scaffolding using the stand-alone scaffolding tool SSPACE (ver. 2.0) with the following parameters: -x 0, -k 3, -a 0.8, and -T 60 [11].”

12. Page 5: "After scaffolding, the number of scaffolds decreased from 153,398 to 12,381, and the N50 scaffold length increased from 6,135 to 166,726 bp (Table 2)."
The authors might consider stating the total size of the final scaffolds (~795 Mb), which is approaching the genome size according to the K-mer analysis.

We added this sentence at the end of paragraph:
“The total size of the final scaffolds (~795 Mb) was consistent with the estimated genome size (805 Mb).”

13. Page 6: "We first identified repetitive elements using RepeatMasker (ver. 3.3.0) [13], and this masked genome sequence was used for ab initio gene prediction using the SNAP software [14]"
Can the authors please provide more details on their use of RepeatMasker? Which repeats were used? How were they generated bioinformatically?

We changed manuscript as follow:
“MAKER2 annotation pipeline was used for genome annotation with default parameters [12]. It first identified repetitive elements using RepeatMasker (ver. 3.3.0) with a *de novo* repeat library [13], which was constructed using RepeatModeler (Ver. 1.0.3) [14] with the Repbase library (Ver. 20140131). The SNAP gene finder [15] was selected to perform *ab initio* gene prediction from this masked genome sequence.”

14. Page 6: "Transcriptome assembly results, which were generated using CLC Genomics Workbench 8.0, were used for expressed sequence tags"
Some more details are needed here. Can the authors please clarify the information in terms of the parameters used in CLC? Also, was there not a step to go from a raw transcriptome to a reference transcriptome assembly used for annotation?

We changed manuscript as follow:
“Transcriptome assembly was performed by using the program CLC Genomics Workbench 8.0 with default parameters, and sequencing reads from PE500 (Table 1) were used.”

15. Page 6: "A total of 32,712 genes were predicted in P. charcoti using MAKER, and 61,709 ab initio

prediction, with insufficient evidence were generated (Table 2)." Much of the information listed in the text is not linked to Table 2. Can the authors please check they have included all information intended in Table 2?

We deleted the ab initio prediction in manuscript and added more information into Table 2.

16. Page 7: Minor point - consider using the term 'partial' rather than 'Fragmented' in Table 1, to be better aligned to information given in the text (or used 'fragmented' in the text). Would the authors also like to comment on why the number of vertebrate BUSCO genes is substantially lower than the eukaryotic or metazoan set?

We change “fragmented” with “Partial” in Table 3. We did re-run BUSCO analysis to the Actinopterygii DB, and changed Table 3 with new results as reviewer’s comments.

17. Page 8: "We identified 8,951 orthologous groups common to all seven fish; 288 of 32,636 N. coriiceps genes and 333 of 32,712 P. charcoti genes were not identified in any other species, and 2,519 groups were identified only in the two Antarctic fish (Fig. 3A). Subsequently, gene gain-and loss was analyzed in seven representative fish species, P. charcoti gained 937 and lost 1916 gene families (Fig. 3B)." The authors must provide methods to explain how the phylogenetic tree provided in Figure 3 was produced and how they performed the gene gain/loss approach. I suspect the methods are the same as presented in Shin et al. 2014, but this should be clarified. I also must request that the authors either directly provide (or offer some easy way) for an interested reader to extract the relevant subsets of the 8,951 orthogroups (e.g. 333 genes specific to P. charcoti; 258 genes specific to N. coriiceps; 2,519 common to the two Antarctic fish) as these will be a useful start point for future investigations. Looking at the current data provided in the GigaDB repository, I can only see the 8,951 orthogroups.

The method producing a “gain-and-loss” was added to manuscript, and we uploaded additional orthogroups data into GigaDB (orthologues_List_specific_Antarctic_fish.txt)

18. Page 10: "Availability of supporting data". The authors should break down the full set of data attached in the GigaDB online repository.

We mended as comment.

Click here to download Personal Cover
coverletter_revision.docx

May 00, 2017

Dear Dr. Hans Zauner, Editor of GigaScience,

We would like to thank you and all the reviewers for your kind help to revise our manuscript and consider our manuscript for publication in GigaScience.

As reviewer comment, we corrected manuscript and added new sentences for revised manuscript. The corrected points were marked in blue color in revised manuscript; "Antarctic dragonfish-R1_ADH-fixed_02_Plain text.docx". Please refer to rebuttal letter "revision_R1_ADH-fixed_02_Plain text.docx" for response to reviewers.

Our changed data files were loaded in GigaDB as folder " Revision01_Data for GigaScience manuscript GIGA-D-17-00041 ". Please refer to the text file "README for Revision01(List of changed file).txt" for list of changed files.

Hope the revised is acceptable for publication. We look forward to hearing your decision.

Thank you very much for your consideration of this paper,

Hyun Park,

Hyun Park, Ph. D.
Korea Polar Research Institute
26 Songdomirae-ro, Yeonsu-gu,
Incheon 406-840, South Korea
Tel: +82-32-760-5570/Fax: +82-32-760-5575
e-mail: hpark@kopri.re.kr

 Click here to download Personal Cover README for Revision01(List of changed file).txt

Supporting data for "Draft genome of the Antarctic dragonfish, Parachaenichthys charcoti"
===

Ahn, D, H; Shin, S, C; Kim, B, M; Kang, S; Kim, J, H; Ahn. I; Park, J; Park, H
(2017) GigaScience Database.

Summary

The Antarctic bathydraconid dragonfish, Parachaenichthys charcoti, is an Antarctic notothenioid teleost endemic to the Southern Ocean.
The Southern Ocean has cooled to ?1.8C over the past 30 million years, and the seawater had retained cold temperature and isolated oceanic environment by Antarctic Circumpolar Current (ACC).
Notothenioids dominate Antarctic fish, making up 90% biomass and all notothenioids have undergone molecular and ecological diversification to survive in this cold environment.
Therefore, they are considered an attractive Antarctic fish model for evolutionary and ancestral genomic studies.
Bathydraconidae is a speciose family of the Notothenioidei, the dominant taxonomic component of Antarctic teleosts.
To understand the process of evolution of Antarctic fish, we select a typical Antarctic bathydraconid dragonfish, P. charcoti.
Here, we have sequenced, de novo assembled and annotated a comprehensive genome from P. charcoti.

The draft genome of P. charcoti is 709 Mb in size.
The N50 contig length is 6,145 bp and its N50 scaffold length 178,362 kb.
The genome of P. charcoti is predicted to contain 32,712 genes, 18,455 of which have been assigned preliminary functions.
A total of 8,951 orthologous groups common to seven species fish were identified, while 333 genes were identified in P. charcoti only; 2,519 orthologous group were also identified in both P. charcoti and N. coriiceps, another Antarctic fish.
Four gene ontology (GO) terms were statistically overrepresented among the 333 genes unique to P. charcoti, according to GO enrichment analysis.

The draft P. charcoti genome will broaden our understanding of the evolution of Antarctic fish in their extreme environment.
It will provide a basis for further investigating the unusual characteristics of Antarctic fishes.

sequence data deposited with the SRA

BioProject : PRJNA330735
 Genomic and transcriptomic sequence data

(1) BioSample: SAMN05421612

muscle from Parachaenichthys charcoti, genomic DNA

(2) BioSample: SAMN05421683
liver sample from Parachaenichthys charcoti, genomic DNA

(3) BioSample: SAMN06232533
liver sample from Parachaenichthys charcoti, transcriptome

Files
-----

(1) PC-genome_assembly.fasta
genome assembly file (fasta)

(2) PC-transcriptome_assembly.fasta
transcriptome assembly file (fasta)

(3) PC-coding gene annotations.gff3
coding gene annotations (gff3)

(4) PC-coding gene nucleotide sequences.fasta
coding gene nucleotide sequences (fasta)

(5) PC-coding gene translated sequences.fasta
coding gene translated sequences (fasta)

(6) PC-repeatmasker.gff3
repeats annotations (gff3)

(7) PC-snap.gff3
snap annotations (gff3)

(8) Blast2Go_annotation_with_AED.tab [changed file]
blast2Go annotation results with AED value (tab)

(9) multi-fasta_alignments_orthologues.zip
Zip file of orthologous gene family alignments (multi-fasta)

(10) multi-fasta_alignments_orthologues List.txt
Summarized list of orthologous gene family alignments

(11) BUSCO_Actinopterygii_report.txt [changed file]
summarized BUSCO output report in the Actinopterygii lineage dataset

(12) orthologues_List_specific_Antarctic_fish.txt [new added file]
orthologues list in Antarctic fish

(13) Phylogenetic Tree.nwk
description of a rooted and bifurcating phylogenetic tree

(14) README.txt [changed file]
including all file names with a brief description of each
