# Supplementary material for: Draft genome of the Antarctic dragonfish, Parachaenichthys charcoti
Source: Gigascience. 2017 Jul 24;6(8):1–6. doi: 10.1093/gigascience/gix060 (PMC5597851; doi:10.1093/gigascience/gix060)
Supplement: Reviewer-1-Report-(Original-Submission).pdf [file gix060_reviewer-1-report-(original-submission).pdf]

Reviewer Report

Title: Draft genome of the Antarctic dragonfish, Parachaenichthys charcoti

Version: Original Submission **Date:** 3/15/2017

Reviewer name: Martin Malmstrøm

Reviewer Comments to Author:

The manuscript "Draft genome of the Antarctic dragonfish, Parachaenichthys charcoti" is another important contribution to the scientific community working on comparative teleost genomics. As with similar studies, this manuscript appears to be submitted with the purpose of releasing this valuable dataset to the public, and holds no claim to solve any specific scientific question, but rather put up possibilities for the future use of this dataset.### Major commentsThe authors have made a good attempt to conduct thorough sequencing of the Antarctic dragonfish genome, using several paired-end and mate-pair libraries. However, the results, and especially the N50 contig statistic is far below what this reviewer would expected using Celera Assembler (CA) with the sequencing data presented. This reviewer is curious to why this specific sequencing method was applied (i.e three very similar libraries for PE sequencing and 2x300bp).For all the paired-end libraries the inserts are shorter than the sequencing output, which appears to be quite wasted as the trimmed reads are only 173-212bp on average for these libraries. Would it not have been better to have libraries with an insert size around 700-800bp? This would surely span many more of the repetitive sequences now causing gaps and low continuity.Also, as trimming is part of the CA pipeline, why trim the reads prior to running CA? Additionally, FLASH should have been applied to merge overlapping reads from the paired-end sequencing libraries prior to assembly.The authors have also made a fair attempt to annotate this P. charcoti draft genome using the MAKER pipeline, and I'm happy to see that effort has been put into RNA sequencing to improve this analysis. However, some shortcuts have been taken in regard to how the annotation was performed. For instance, it is now standard procedure to produce a species specific repeat library, using RepeatModler to aid in the annotation. This was not done. The authors also fail to inform which library that was used for identifying repetetive elements with RepeatMasker. It is also customary to include SNAP, AUGUSTUS and GENEFINDER runs as part of the MAKER pipeline to improve gene prediction. This reviewer cannot see that this has been included in the annotation pipeline, which might explain why the number of predicted genes is so high. I'm also missing information regarding which AED cut-off that was used for the final gene predictions.The authors have further investigated the gene space completeness using BUSCO, which is good. However, there is reasons to belive that the gene sets reported are not up to date, especially since there is now a Actinopterygii specific gene set available (http://busco.ezlab.org/frame_meta.html). This should be quick to run and the results can easily be implemented in Table 3.In an attempt to conduct comparative genomics, the authors have grouped orthologous genes from several species into orthologous groups using OrthoMCL. This is an OK starting point for a comparative analysis, however, their analysis is based on unfiltered data for the ENSEMBLE (which is know to include thousands of duplicates and Gene ID's without any sequence data available). For instance, would 24,460 genes be a much more adequate dataset to use for the zebrafish. It also

included all of the 32,712 P. charcoti gene predictions, which leads me to belive that most of the 333 othologous groups (according to Figure 3a, yet referred to as 333 genes" in the text) contain false positives and/or repeats. Based on these results, the authors also produce a "gain-and-loss" figure for the investigated species, yet there is no mentioning on how this analysis was performed.Finally, the authors also present analyses based on (crude) Gene Ontology analyses which offer little scientific value. The entire paragraph on GO enrichment testing (including the results) it not very interesting. So, unless there is any biological meaning applied to the genes or pathways identified, this could/should be removed.### Minor commentsi) Please use an appropriate "thousands seperator" for all values across the manuscriptii) Please make sure that the genus name is not spelled out several times.iii) Excange "illumine" for "Illumina" prior to Table 1

Level of Interest

Please indicate how interesting you found the manuscript: An article of importance in its field

Quality of Written English

Please indicate the quality of language in the manuscript: Acceptable

Declaration of Competing Interests

Please complete a declaration of competing interests, considering the following questions:

- Have you in the past five years received reimbursements, fees, funding, or salary from an organisation that may in any way gain or lose financially from the publication of this manuscript, either now or in the future?
- Do you hold any stocks or shares in an organisation that may in any way gain or lose financially from the publication of this manuscript, either now or in the future?
- Do you hold or are you currently applying for any patents relating to the content of the manuscript?
- Have you received reimbursements, fees, funding, or salary from an organization that holds or has applied for patents relating to the content of the manuscript?
- Do you have any other financial competing interests?
- Do you have any non-financial competing interests in relation to this paper?

If you can answer no to all of the above, write 'I declare that I have no competing interests' below. If your reply is yes to any, please give details below.

I declare that I have no competing interests

I agree to the open peer review policy of the journal. I understand that my name will be included on my report to the authors and, if the manuscript is accepted for publication, my named report including any

attachments I upload will be posted on the website along with the authors' responses. I agree for my report to be made available under an Open Access Creative Commons CC-BY license (http://creativecommons.org/licenses/by/4.0/). I understand that any comments which I do not wish to be included in my named report can be included as confidential comments to the editors, which will not be published.

I agree to the open peer review policy of the journal
