# Supplementary material for: Draft genome of the Antarctic dragonfish, Parachaenichthys charcoti
Source: Gigascience. 2017 Jul 24;6(8):1–6. doi: 10.1093/gigascience/gix060 (PMC5597851; doi:10.1093/gigascience/gix060)
Supplement: Reviewer-1-Report-(Revision-1).pdf [file gix060_reviewer-1-report-(revision-1).pdf]

**Reviewer Report**

**Title:** Draft genome of the Antarctic dragonfish, Parachaenichthys charcoti

**Version:** Revision 1 **Date:** 5/30/2017

**Reviewer name:** Martin Malmstrøm

**Reviewer Comments to Author:**

This reviewer is happy to accept the manuscript for publication as the raised concerns have beed adresses and or clarified. Minor language corrections (grammar) still needs to be addressed though. Having a native English speaker to look over the manuscript before resubmission would certainly improve the over all impression of this work.

**Level of Interest**

Please indicate how interesting you found the manuscript: An article of importance in its field

**Quality of Written English**

Please indicate the quality of language in the manuscript: Needs some language corrections before being published

**Declaration of Competing Interests**

Please complete a declaration of competing interests, considering the following questions:

- Have you in the past five years received reimbursements, fees, funding, or salary from an organisation that may in any way gain or lose financially from the publication of this manuscript, either now or in the future?
- Do you hold any stocks or shares in an organisation that may in any way gain or lose financially from the publication of this manuscript, either now or in the future?
- Do you hold or are you currently applying for any patents relating to the content of the manuscript?
- Have you received reimbursements, fees, funding, or salary from an organization that holds or has applied for patents relating to the content of the manuscript?
- Do you have any other financial competing interests?
- Do you have any non-financial competing interests in relation to this paper?

If you can answer no to all of the above, write 'I declare that I have no competing interests' below. If your reply is yes to any, please give details below.

I declare that I have no competing interests

I agree to the open peer review policy of the journal. I understand that my name will be included on my report to the authors and, if the manuscript is accepted for publication, my named report including any attachments I upload will be posted on the website along with the authors' responses. I agree for my report to be made available under an Open Access Creative Commons CC-BY license (http://creativecommons.org/licenses/by/4.0/). I understand that any comments which I do not wish to be included in my named report can be included as confidential comments to the editors, which will not be published.

I agree to the open peer review policy of the journal
