# Supplementary material for: Draft genome of the Antarctic dragonfish, Parachaenichthys charcoti
Source: Gigascience. 2017 Jul 24;6(8):1–6. doi: 10.1093/gigascience/gix060 (PMC5597851; doi:10.1093/gigascience/gix060)
Supplement: Reviewer-2-Report-(Original-Submission).pdf [file gix060_reviewer-2-report-(original-submission).pdf]

Reviewer Report

Title: Draft genome of the Antarctic dragonfish, Parachaenichthys charcoti

Version: Original Submission **Date:** 3/28/2017

Reviewer name: Daniel Macqueen

Reviewer Comments to Author:

Review of Manuscript GIGA-D-17-00041

Overview

Hyun Park's group present the first genome sequence for Parachaenichthys charcoti, a member of the bathydraconid (Antarctic dragonfish) clade of the notothenioid group of Antarctic teleosts. This is the second notothenioid genome to be made publically available, following the publication of the Antarctic bullhead Notothenia coriiceps (Shin SC et al. Genome Biology. 2014;15:468). As a fish biologist interested in physiological evolution, the availability of multiple notothenioid genomes presents a great opportunity for deciphering the genomic basis of adaptive/non-adaptive changes made possible by the extreme cold environment and unusual evolutionary history linked to the notothenioid radiation. As a resource, the P. charcoti genome will be used for comparative analyses with N. coriiceps and other teleost genomes. I am particularly excited about the eventual publication of a genome for an Antarctic icefish species (Channichthyidae), for which the most extreme physiological traits linked to cold conditions are observed (e.g. total loss of haemoglobin). The genomes of N. corriceps and P. charcoti will be crucial for such comparative analyses. It is important to note that the Antarctic dragonfishes and Notothenia lineages are relatively distant, so the availability of both genome sequences allows both shared-ancestral and lineage-specific changes or adaptations to be disentangled. Moreover, these genomes are generally important in the context of understanding the physiological capacity of notothenioids - key to the overall fauna of Antarctica - to respond to contemporary changes in climate. The manuscript is generally well written.

Thus, overall, I support the publication of this Data Note in GigaScience and I think the paper will encourage the uptake of the P. charcoti genome for a range of physiological and evolutionary questions. The data provided by the authors is generally comprehensive and relevant. I offer a number of comments/suggestions, aiming to either increase the clarity surrounding the manuscript's organization and the data and its applications, or requesting more details on aspects of the methodology. I split my comments into general suggestions and a larger set of minor points, the latter linked to particular text in the paper.

General suggestions

1. The authors might consider adding an informative heading to the first paragraph of the Data

Description section, such as 'Context' or "Background". This would increase the clarity of the manuscript's organization.

2. I suggest that authors include an additional dedicated section at the end of the manuscript along the lines of the "Re-use potential" subheading suggested in the Journal guidelines. At the moment, the paper does not do a very proficient job in helping the reader envisage specific uses for the Data Set presented. Hence, in current form, the wider interest of the data set is not fully justified. I would like to see elaboration of the author's stance concerning data re-use, which I feel is necessary to meet the Journal's aim to "contextualize exceptional datasets to encourage reuse". This could provide more context in light of the findings of Shin et al. 2014 (e.g. the new P. charcoti genome will allow questions such as, which genomic traits are ancestral to all notothenioids? Which are lineage-specific? Which evolved by convergence? etc.), or give more context on interesting physiological traits observed in notothenioids for which researchers are seeking to clarify the underlying genomic basis.

3. I find the GO analyses to have tangential relevance as a dataset of meaningful future use, unless it is dissected considerably more than presented within this Data Note, where it appears much as a 'bolt-on'. The biological meaning of data presented in Table 4 (overrepresented GO terms in P. charcoti) does not add much insight to fuel on-going research. The data in Table 5 may be misleading in terms of its potential meaning for notothenioid-specific evolution, since the gene list was defined on the basis of comparing two notothenioids with stickleback as the next nearest phylogenetic lineage. As tens of millions of years separate notothenioids and stickleback, it is impossible to say the genes are restricted to notothenioids. This is a minor point, but for me, the paper would be clearer without the GO analyses.

4. The authors should use species abbreviations consistently throughout the manuscript, which is not the case currently.

5. The authors used Celera to assemble the paired end MiSeq reads. As this is an OLC assembler, I would not have naturally considered this to be an optimal approach using relatively short read lengths provided by MiSeq. However, the authors provide evidence that a reasonable draft genome and annotation was nonetheless generated. I am intrigued, in a comparative sense, to know how the same data would have performed using the best-performing assemblers built on the de Bruijn Graph approach. Did the authors attempt any such assemblies, and if so, why did they eventually choose to go with the Celera assembly? To clarify, I am not requesting this as a necessary revision, though if the authors had some available data, I feel it would be of wider interest to contrast the performance of different assemblers.

Specific minor points

1. Abstract: "… and P. charcoti has undergone molecular and ecological diversification to survive in this cold environment". The wording here can be misconstrued, as the same statement is true for the wider notothenioid lineage. Better to write "… and all notothenioids have undergone molecular and ecological diversification to survive in this cold environment.

2. "However, little is known about the biology of this species, except that globin intergenic regulatory regions play a role in its low levels of alpha-globin expression". I found this sentence a little disappointing as an upfront motivation for the Data. I feel the abstract could more strongly communicate the importance of the target species for our comparative understanding of evolution in Antarctic fish. Which genomic traits are ancestral to notothenioids, which are lineage-specific, which evolved by convergence, etc.? I suspect these are the motivating questions and in my opinion, the paper would be stronger if this came through more strongly generally, including the abstract.

3. Keywords: the authors might consider elaborating this list, for example to include mention of a genome assembly. Currently the keyword list could be linked to almost any field where Antarctic fish are studied, so it should better represent a genome biology paper.

4. Data description paragraph 1: "Antarctic notothenioid teleosts have evolved to adapt to the extreme Antarctic marine environment. The fish fauna of the Southern Ocean is dominated by a single lineage belonging to the perciform suborder Notothenioidei, consisting of 132 species and 8 families. They survive in the extreme Antarctic marine environment, which includes large seasonal changes in food availability and cold ocean water.

These first few sentences have an issue with the flow of information, which jumps about abruptly, as if thrown together. Consider a reformulation: "The fish fauna of the Southern Ocean is dominated by a single lineage belonging to the perciform suborder Notothenioidei, consisting of 132 species and 8 families. All Antarctic notothenioids have evolved to adapt to the extreme Antarctic marine environment, which includes large seasonal changes in food availability and stably cold water temperature."

5. Data description paragraph 1: "Nototheniidae is the most speciose family of the Notothenioidei, the dominant taxonomic component of Antarctic teleosts, making up 90% of the fish biomass of the continental shelf and upper slope [1-4]. Parachaenichthys charcoti, the Antarctic bathydraconid dragonfish, was first described by Vaillant in 1906".

I find the construction of these sentences to be unusual - when first reading the information, the implication I got was that P. charcoti is a member of Nototheniidae, which is not the case. Can the authors please address the construction of the text to improve the clarity of the information?

6. Page 4, "All sequencing …. (Table 1)", would read more clearly as "All sequencing …. (data statistics provided in Table 1)". In the current form, the table citation is not clearly linked to the provided text about 'sequencing processes'.

7. Page 4, "illumine, Carlsbad, USA". Please correct the typo.

8. Page 4, "Finally, paired-end trimmed reads data with 73-fold coverage were obtained (Table 1).

How was the fold-coverage estimated in this case? Also, why present coverage just for the paired-end libraries and not the mate pair libraries?

9. Page 5: "The assembled contig revealed a contig coverage of approximately 36.57x". By what approach was this assessed?

10. Page 5: Why were the selected parameters in Celera selected? Are these simply generally optimized default parameters?

11. Page 5: "Contigs from the initial assembly were used for scaffolding using the stand-alone scaffolding tool SSPACE (ver. 2.0) [11]. Trimmed mate-pair reads created using the FASTX-Toolkit were used in the scaffolding process".

Can the authors please provide enough information on the SSPACE parameters employed to allow the reader to repeat the analysis?

12. Page 5: "After scaffolding, the number of scaffolds decreased from 153,398 to 12,381, and the N50 scaffold length increased from 6,135 to 166,726 bp (Table 2)."

The authors might consider stating the total size of the final scaffolds (~795 Mb), which is approaching the genome size according to the K-mer analysis.

13. Page 6: "We first identified repetitive elements using RepeatMasker (ver. 3.3.0) [13], and this masked genome sequence was used for ab initio gene prediction using the SNAP software [14]"

Can the authors please provide more details on their use of RepeatMasker? Which repeats were used? How were they generated bioinformatically?

14. Page 6: "Transcriptome assembly results, which were generated using CLC Genomics Workbench 8.0, were used for expressed sequence tags"

Some more details are needed here. Can the authors please clarify the information in terms of the parameters used in CLC? Also, was there not a step to go from a raw transcriptome to a reference transcriptome assembly used for annotation?

15. Page 6: "A total of 32,712 genes were predicted in P. charcoti using MAKER, and 61,709 ab initio prediction, with insufficient evidence were generated (Table 2)."

Much of the information listed in the text is not linked to Table 2. Can the authors please check they have included all information intended in Table 2?

16. Page 7: Minor point - consider using the term 'partial' rather than 'Fragmented' in Table 1, to be better aligned to information given in the text (or used 'fragmented' in the text). Would the authors also like to comment on why the number of vertebrate BUSCO genes is substantially lower than the eukaryotic or metazoan set?

17. Page 8: "We identified 8,951 orthologous groups common to all seven fish; 288 of 32,636 N. coriiceps genes and 333 of 32,712 P. charcoti genes were not identified in any other species, and 2,519 groups were identified only in the two Antarctic fish (Fig. 3A). Subsequently, gene gain-and loss was analyzed in seven representative fish species, P. charcoti gained 937 and lost 1916 gene families (Fig. 3B)."

The authors must provide methods to explain how the phylogenetic tree provided in Figure 3 was produced and how they performed the gene gain/loss approach. I suspect the methods are the same as presented in Shin et al. 2014, but this should be clarified. I also must request that the authors either directly provide (or offer some easy way) for an interested reader to extract the relevant subsets of the 8,951 orthogroups (e.g. 333 genes specific to P. charcoti; 258 genes specific to N. coriiceps; 2,519 common to the two Antarctic fish) as these will be a useful start point for future investigations. Looking at the current data provided in the GigaDB repository, I can only see the 8,951 orthogroups.

18. Page 10: "Availability of supporting data". The authors should break down the full set of data attached in the GigaDB online repository.

Level of Interest

Please indicate how interesting you found the manuscript: An article of importance in its field

Quality of Written English

Please indicate the quality of language in the manuscript: Acceptable

Declaration of Competing Interests

Please complete a declaration of competing interests, considering the following questions:

- Have you in the past five years received reimbursements, fees, funding, or salary from an organisation that may in any way gain or lose financially from the publication of this manuscript, either now or in the future?
- Do you hold any stocks or shares in an organisation that may in any way gain or lose financially from the publication of this manuscript, either now or in the future?
- Do you hold or are you currently applying for any patents relating to the content of the manuscript?

- Have you received reimbursements, fees, funding, or salary from an organization that holds or has applied for patents relating to the content of the manuscript?
- Do you have any other financial competing interests?
- Do you have any non-financial competing interests in relation to this paper?

If you can answer no to all of the above, write 'I declare that I have no competing interests' below. If your reply is yes to any, please give details below.

I declare that I have no competing interests

I agree to the open peer review policy of the journal. I understand that my name will be included on my report to the authors and, if the manuscript is accepted for publication, my named report including any attachments I upload will be posted on the website along with the authors' responses. I agree for my report to be made available under an Open Access Creative Commons CC-BY license (http://creativecommons.org/licenses/by/4.0/). I understand that any comments which I do not wish to be included in my named report can be included as confidential comments to the editors, which will not be published.

I agree to the open peer review policy of the journal
